# Rewiring of Molecular Networks Induced by the Combination of Loratadine, Raloxifene, and Sorafenib Leads to the Identification of Clinically Relevant Therapeutic Targets in Hepatocellular Carcinoma

**DOI:** 10.3390/biomedicines14091898

**Published:** 2026-08-25

**Authors:** Fernanda Villarruel-Melquiades, Nancy Santos-Martínez, Martha Noyola-Díaz, Estefanía de Jesús Terán-Sánchez, José Iván Serrano-Contreras, Luis Gerardo Zepeda-Vallejo, María Eugenia Mendoza-Garrido, Julio Isael Pérez-Carreón, Cecilia Bañuelos, Georgina Hernández-Montes, Javier Camacho

**Affiliations:** 1 Departamento de Farmacología, Centro de Investigación y de Estudios Avanzados del Instituto Politécnico Nacional, Av. Instituto Politécnico Nacional 2508, Ciudad de Mexico CP 07360, Mexico; junoyola@cinvestav.mx; 2 Independent Researcher, CP 33000 Bordeaux, France; santos_2105@hotmail.com; 3 Departamento de Química Orgánica, Escuela Nacional de Ciencias Biológicas, Instituto Politécnico Nacional, Prolongación de Carpio y Plan de Ayala s/n, Col. Santo Tomas, Alcaldía Miguel Hidalgo, Ciudad de Mexico CP 11340, Mexico; estefaniateran92@gmail.com (E.d.J.T.-S.); luisgzepeda@gmail.com (L.G.Z.-V.); 4 Department of Metabolism, Digestion and Reproduction, Section of Nutrition, Faculty of Medicine, Commonwealth Building Hammersmith Campus, Imperial College London, London W12 0NN, UK; j.serrano-contreras@imperial.ac.uk; 5 Departamento de Fisiología, Biofísica y Neurociencias, Centro de Investigación y de Estudios Avanzados del Instituto Politécnico Nacional, Av. Instituto Politécnico Nacional 2508, Ciudad de Mexico CP 07360, Mexico; mmendoza@fisio.cinvestav.mx; 6 Instituto Nacional de Medicina Genómica (INMEGEN), Periférico Sur No. 4809, Col. Arenal Tepepan, Ciudad de Mexico CP 14610, Mexico; jiperez@inmegen.gob.mx; 7 Programa Transdisciplinario en Desarrollo Científico y Tecnológico para la Sociedad, Departamento de Investigación y Estudios Multidisciplinarios, Centro de Investigación y de Estudios Avanzados del Instituto Politécnico Nacional, Av. Instituto Politécnico Nacional 2508, Ciudad de Mexico CP 07360, Mexico; cebanuelos@cinvestav.mx; 8 Red de Apoyo a la Investigación, Coordinación de Investigación Científica, Universidad Nacional Autónoma de Mexico, Ciudad de Mexico CP 14080, Mexico; yinna@cic.unam.mx

**Keywords:** hepatocellular carcinoma, multi-omics, drug repurposing, drug combination, loratadine, raloxifene, sorafenib

## Abstract

**Background/Objectives**: Hepatocellular carcinoma (HCC) is the most prevalent primary liver tumor and is often diagnosed at advanced stages with very poor therapeutic response, leading to high mortality. Thus, new therapeutic strategies and biomarkers are urgently needed. We previously showed that the combination of loratadine, raloxifene, and sorafenib exerts synergistic cytotoxicity on HCC cells. Here, we explored potential molecular mechanisms underlying the anticancer effects of this combination using multiomics analyses. **Methods**: We performed proteomic analyses based on mass spectrometry, transcriptomic analyses using the Clariom D Plus human microarray (Affymetrix), and metabolomic analyses based on nuclear magnetic resonance to investigate the profile changes induced by the drug combination in HuH7 cells. Bioinformatic analyses were applied to associate the omics changes with biological functions, molecular interactions, and clinical relevance in terms of patient survival. **Results**: We identified several molecules whose expression changed in response to treatment across the three omics profiles analyzed. Some of them were found to be involved in hallmarks of cancer, including sustained proliferation, evasion of growth suppressors, and resistance to cell death. Integrated multi-omics analyses revealed that the drug combination suppresses critical oncogenic drivers (C7orf50, NUP188, and HS2ST1) and that the mitotic cell cycle process, DNA synthesis and cholesterol biosynthesis are the primary pathways affected. Protein–protein interaction analysis revealed five key hubs (KIF2C, PCNA, TRIP13, NDC80, and RPA3), whose expression in HCC is associated with poor clinical prognosis. **Conclusions**: The combined treatment rewired molecular networks involved in HCC progression. These findings identify clinically relevant molecular targets associated with poor prognosis and provide mechanistic insights into the synergistic anticancer activity of this drug combination.

## 1. Introduction

Liver cancer ranks eighth in incidence and fourth in mortality worldwide [1]. Hepatocellular carcinoma (HCC) is the most common primary liver tumor, accounting for 85–90% of diagnosed cases [2]. This cancer commonly occurs in the context of chronic liver disease, and disorders such as cirrhosis increase the risk of developing this malignant tumor [3]. Although the hepatitis B vaccine reduces the risk of developing HCC, hepatitis B virus infection remains the leading risk factor for this cancer for approximately 50% of cases [3,4].

Sorafenib is a first-line pharmacological treatment option for patients with advanced HCC [5]. However, a large percentage of these patients do not benefit clinically in the long term due to acquired resistance to the drug [6,7]. This therapeutic failure is driven by a complex multifactorial network that includes the compensatory activation of parallel oncogenic signaling pathways (such as PI3K/Akt/mTOR, Wnt/β-catenin, and EGFR/FGFR), metabolic reprogramming that evades cell death processes such as ferroptosis and autophagy, alterations in the tumor microenvironment resulting from hypoxia and immunosuppression, and cellular plasticity associated with the epithelial-to-mesenchymal transition (EMT) and the acquisition of cancer stem cell properties, among others [7,8].

The therapeutic targets of sorafenib include serine/threonine and tyrosine kinases such as VEGF, PDGF, RAF kinases, c-KIT, FLT3 and BRAF. Inhibition of these kinases results in reduced angiogenesis and cell proliferation while inducing apoptosis in tumor cells [9].

The discovery of key molecules in liver carcinogenesis is a powerful and promising strategy for identifying new therapeutic options. In fact, many studies have focused on understanding the molecular mechanisms involved in HCC development to identify better treatment options. Unfortunately, key driver mutations in HCC, including those in *TERT*, *TP53*, and *CTNNB1*, remain undruggable [10].

Given the limited efficacy and high rate of resistance associated with current therapies for HCC, there is an urgent need to identify more effective therapeutic strategies. In this context, drug repurposing has emerged as a highly attractive approach in oncology. For example, accumulating preclinical and clinical evidence highlights antihistamines, such as loratadine, as a promising candidate for cancer therapy due to their modulatory effects on tumor growth, cell survival, induction of apoptosis, and others [11]. This H1 antihistamine has been shown to exhibit significant antitumor potential; in lung adenocarcinoma, loratadine inhibits proliferation and promotes cell death [12,13]. Similarly, in breast cancer models, its co-administration in nanostructures enhances the antimitotic activity of conventional chemotherapeutic agents such as 5-fluorouracil, reducing the required doses [14]. On the other hand, raloxifene—a second-generation selective estrogen receptor modulator (SERM)—has demonstrated significant antitumor potential through both estrogen receptor-dependent and -independent mechanisms. Scientific evidence highlights its ability to inhibit tumor proliferation and invasion by modulating key oncogenic signaling pathways, such as the IL-6/STAT3 cascade, and to induce cell cycle arrest and apoptosis in various tumor models [15,16]. Furthermore, the characterization of their pharmacological profile and the development of structural analogs support their efficacy in disrupting cell survival mechanisms and overcoming resistance to conventional therapies [17]. Thus, the complementary action of both drugs supports the importance of integrating loratadine and raloxifene into repositioning strategies targeting complex molecular networks in oncology.

To overcome these complex resistance networks and achieve potential significant benefits for patients, it has been essential to move from single-agent-based paradigms to rational combinations targeting multiple molecules, which led us to propose a drug combination. In the search for new pharmacological strategies against HCC, we focused on a drug repurposing approach using raloxifene and loratadine, both of which have reported independent antineoplastic effects. Raloxifene, a selective estrogen receptor modulator (SERM), interferes with tumor progression by inhibiting key cell proliferation and survival pathways, such as IL-6/GP130, JAK2/STAT3, and AKT. Meanwhile, the antihistamine loratadine has been epidemiologically associated with reduced mortality in cancer patients, demonstrating capabilities to induce lysosomal damage, block TRPV2 calcium channels, and inhibit the NF-κB/AP-1 pathway [18]. Consequentially, we evaluated the effect of co-incubating human HCC cell lines with the combination of loratadine, raloxifene, and sorafenib (LRS). In summary—at the molecular level—the rationale for the use of the LRS combination relies on a multi-targeted mechanism designed to overcome the single-drug limitations and resistance development in HCC. Sorafenib primarily inhibits tyrosine kinases, thereby suppressing the survival and proliferation of tumor cells. In addition, raloxifene acts as a selective estrogen receptor modulator that attenuates the oncogenic activation of STAT3/Akt, while promoting endoplasmic reticulum stress. Finally, loratadine alters lysosomal membrane permeability and autophagic flux, compromising cellular proteostasis. By simultaneously interfering with these molecular mechanisms, the LRS combination may disrupt compensatory survival mechanisms and induce severe, irreversible cellular stress, ultimately leading hepatoma cells to cell death.

We found that this combination synergistically induced cytotoxicity in the cells [19]. The safety of some of these drugs and the significant antiproliferative effects of this combination, make it a promising approach for HCC patients. Therefore, elucidating the potential molecular mechanisms underlying the cytotoxicity of the LRS combination may provide very important insights into the discovery of novel anticancer targets. To this end, we performed an integrative analysis of the transcriptome, proteome, and metabolome profiles in HuH7 cells before and after LRS treatment. Our findings indicate that this multi-drug combination reprograms molecular networks with a potential clinical relevance in HCC. Moreover, this multi-omics analysis not only provides insights into the synergistic antiproliferative effects of these drugs but also unveils molecular components that contribute to liver carcinogenesis.

## 2. Materials and Methods

### 2.1. Cell Culture and Reagents

The human liver cancer cell line HuH7 was obtained from the American Type Culture Collection (ATCC, Manassas, VA, USA) and cultured according to the manufacturer’s instructions. For all omics assays, once the cells reached 80% confluence, they were incubated with either vehicle-only (DMSO) or the combination of loratadine, raloxifene, and sorafenib at their 20% inhibitory concentrations (IC_20_, previously obtained [19]) for 72 h. The drugs were purchased from Sigma Aldrich Co. (St. Louis, MO, USA). To avoid solvent-induced cytotoxicity or physiological alterations, the final concentration of DMSO in all assays was maintained below 0.06%.

Transcriptomic, proteomic and metabolomic analyses were conducted on independent biological replicates generated under strictly standardized, identical experimental conditions, including culture medium, treatment concentration and exposure duration.

### 2.2. Protein Extraction for Proteome Analysis

Protein extraction was performed with three biological replicates for each experimental condition. HuH7 cells were seeded in 10 cm Petri dishes for 72 h in the presence of the vehicle (DMSO) or the drug combination at their IC_20_ [11]. At the end of the incubation period, the cells were centrifuged at 115× *g* for 10 min and the pellet was exposed for 30 min to 500 μL of RIPA lysis buffer (150 mM NaCl, 50 mM Tris, sodium deoxyribonucleotide, 10% Triton, 10% SDS, 200 nM PMSF, 20X Complete Mini, 200 mM orthovanadate, 99.5% glycerol, and 500 mM NaF). After incubation, the samples were centrifuged at 153× *g* for 10 min the samples were centrifuged and stored at −80 °C until further analysis. Protein concentrations were determined using the BCA kit (Cat. No. 23250, Thermo Fisher Scientific, Rockford, IL, USA), and protein integrity was assessed by gel electrophoresis.

### 2.3. Sample Preparation and Mass Spectrometry-Based Proteomics

Proteins were digested and purified using the SP3 method in combination with the iST 8× kit (PreOmics, Münich, Germany), according to the manufacturer’s instructions. Samples containing tryptic peptides were injected in MSE mode in a Synapt G2-Si mass spectrometer (Waters Corp, Milford, MA, USA) to obtain the Area Under the Curve (AUC) and to normalize the amount of tryptic peptides between injections, as a primary quality-control measure, described by Ríos-Castro et al. [20]. The spectral data were acquired using nano-electrospray ionization and ion mobility separation (IMS) with a Full-Scan DIA approach via High-Definition Multiplexed MS/MS (HDMSE) mode [21,22]. As an additional quality-control parameter, system calibration was performed with [Glu1]-Fibrinopeptide fragments via the precursor ion [M+ 2H]2+ = 785.84261, with a fragmentation of 32 eV and an error of less than 1 ppm in all MS/MS measurements.

The *.raw files containing MS and MS/MS spectra were analyzed using Protein Lynx Global Server (PLGS v3.0.3, Waters Crop., Milford, MA, USA) for qualitative identification or Progenesis QI for Proteomics (v4.2, Nonlinear Dynamics, Newcastle, UK) for relative quantitative analysis. Searches were performed against a *Homo sapiens* *.fasta file (downloaded from Uniprot on 27 February 2024 UP0000005640, 104,557 protein sequences) employing a target-decoy strategy to filter false positives [23,24].

Database search parameters and protein identification criteria were defined as follows: trypsin digestion allowing up to 1 missed cleavage; carbamidomethylation (C) as a fixed modification; and N-terminal amidation, deamidation (N, Q), oxidation (M), and phosphorylation (S, T, Y) as variable modifications. Automatic mass tolerances were assigned for peptides and fragments. Identification thresholds were strictly set to a minimum of 2 fragment ion matches per peptide, 5 fragment ion matches per protein, and 1 unique peptide match per protein. Statistical thresholds were controlled by enforcing a maximum False Discovery Rate (FDR) ≤ 1%. All false-positive/reversed decoy matches were systematically excluded prior to exporting final data matrices for statistical analysis.

### 2.4. RNA Extraction for Transcriptome Analysis

For RNA extraction, four biological replicates for each condition were included. HuH7 cells were seeded in 10 cm Petri dishes for 72 h in the presence of the vehicle (DMSO) or the drug combination at their IC_20_. After the incubation period, the cells were treated with TRIzol™ Reagent (Cat. No. 15596018, Thermo Fisher Scientific, Carlsbad, CA, USA) for subsequent RNA extraction. Purity ratios (A_260_/A_280_ and A_280_/A_230_) were measured using a NanoDrop spectrophotometer (Thermo Fisher Scientific, USA). The integrity and quality of the extracted total RNA were evaluated by automated capillary microfluidic electrophoresis using the Agilent 2100 Bioanalyzer system (Agilent Technologies, Santa Clara, CA, USA) and the Eukaryote Total RNA Nano assay kit. Quantitative and qualitative sample analyses were determined based on the electropherogram profile, calculating the ribosomal RNA subunit ratio (28S/18S rRNA) and the RNA Integrity Number (RIN) via the 2100 Expert software. Only samples meeting the minimum quality thresholds defined as RIN > 6.5, 28S/18S rRNA ratio ≥ 1.4, A_260_/A_280_ ≥ 1.6 and A_280_/A_230_ ≥ 2.0 were selected for subsequent expression microarray analyses.

### 2.5. RNA Microarrays

A total of eight hybridization runs were performed on the Affymetrix Clariom D Plus Human Microarray (902923, Affymetrix, Santa Clara, CA, USA). Microarray hybridization and scanning procedures were performed according to the instructions in the Affymetrix Corporation GeneChip Expression Arrays User Guide. The array was scanned using a GeneChip Scanner 3000 7G system (Affymetrix, CA, USA) according to the manufacturer’s instructions, generating data files in ARR, AUDIT, DAT, CEL, and JGP formats.

### 2.6. Extraction of Hydrophilic Metabolites for Metabolome Analysis

The extraction of hydrophilic metabolites was performed with six biological replicates for each experimental condition (DMSO and the LRS combination at their IC_20_) from HuH7 cells. The cells were seeded in 10 cm Petri dishes and treated with DMSO or the LRS combination for 72 h once 80% confluence was reached. Then the culture medium was removed, and metabolites were extracted using a chilled mixture of methanol:chloroform:water (2:2:1.8). The samples were centrifuged at 2600× *g* for 30 min at 4 °C, and the aqueous phase was collected in Eppendorf tubes. The samples were dried in a SpeedVac (Savant DNA120 SpeedVac Concentrator, TermoFisher Scientific, Waltham, MA, USA) and stored at −80 °C until NMR analysis. Water was included as an experimental blank, in addition to the culture media, to identify potential filter-derived contaminants.

The dried samples were resuspended in 800 μL of double-distilled water and filtered (Thermo Scientific™ Titan3™ PTFE Hydrophilic Syringe Filters, 0.2 μm pore, 17 mm diameter; Cat. No. 14-823-300, Thermo Scientific, Waltham, MA, USA). Then, 540 μL of clear sample was mixed with 60 μL of buffer solution (1.5M KH_2_PO_4_, 2mM NaN_3_, 1% TSP (3-(trimethylsilyl)-[2,2,3,3-2H4]-propionic acid sodium salt, Cat. No. 269913, Sigma-Aldrich, St. Louis, MO, USA), 100% D_2_O, pH 7.4). All prepared samples were placed in 5 mm NMR tubes.

### 2.7. ^1^H NMR Spectroscopy Analysis and NMR Data Processing

The ^1^H NMR experiments were conducted using a 600 MHz (14.1 T) spectrometer (Bruker Biospin, Rheinstetten, Germany) equipped with a PA BBO 600S3 BBF-H-D-05 ZSP probe. The samples were measured at 298 K without spinning using the following parameter sets: 4 dummy scans, 32K time domain points, a spectral window of 10 ppm, receiver gain 90.5, 2.18 s acquisition time, 5 s relaxation time, 64 scans, and a digital resolution of 0.45 Hz. All ^1^H NMR spectra were acquired with water suppression using the 1D noesypr1d pulse sequence (Bruker nomenclature, Rheinstetten, Germany).

All 1D ^1^H NMR spectra were manually phased, baseline-corrected, and referenced to the TSPresonance at δ 0.0 using the Topspin 3.6.1 software (BrukerBioSpin, Rheinstetten, Germany). Full resolution 1D ^1^H NMR spectra (∼12K spectral variables) were imported into MatLab (R2014a, The MathWorks, Natick, MA, USA). The spectral regions containing the resonances of residual water (δ 4.2–6.1), TSP (δ 0–0.8), acetone (δ 2.23–2.24, filter-derived contaminant, validated by the analysis of blanks), and noise (δ 8.67–9.5) were removed prior to normalization using the probabilistic quotient method [25]. A single representative peak in the spectra was selected for each identified metabolite, whose areas were estimated using trapezoidal numerical integration and 21 metabolites were obtained using this method. The peak intensities were identified by an extensive and careful inspection/peak-picking procedure allowing that integrals of the corresponding spectral region were the same for all samples. Overlapping resonances were carefully assessed during metabolite annotation using diagnostic chemical shifts, multiplicities, resonance patterns, and comparison with reference spectra acquired from commercial standards. Where substantial overlap could compromise reliable assignment, only well-resolved resonances were considered for area integration. Internal and external databases such as the Human Metabolome Data Base (HMDB; http://hmdb.ca/ accessed on 12 Apr 2026), the Biological Magnetic Resonance Data Bank (BMRB; https://bmrb.io/metabolomics/ accessed on 12 April 2026) or the Chenomx reference library (Chenomx NMR Suite 8.5, Edmonton, AB, Canada), were used for confirmation of metabolite identification.

### 2.8. Bioinformatic Analysis

Prior to downstream bioinformatic analyses, raw datasets from each omics platform were subjected to specific preprocessing, normalization, and scaling pipelines to ensure data quality and comparability. For proteomic analysis, raw protein abundance profiles were processed using the OmicScope platform (https://omicscope.ib.unicamp.br accessed on 3 April 2026), where potential common contaminants were automatically excluded from the dataset. Within the OmicScope pipeline, ‘Progenesis’ was selected as the search engine to import the quantitative protein abundance matrix. Data imputation and sample normalization were set to ‘none’ since the input values had been previously quantified; however, a log-transformation was applied to the protein intensities before statistical analysis. For transcriptomic data, raw microarrays were processed in the Transcriptome Analysis Console (TAC, version 4.0.1, Affymetrix Inc., Santa Clara, CA, USA), where normalization and probe summarization were performed using the RMA algorithm, followed by log_10_ transformation. Metabolomic data normalization, transformation, and scaling were performed using the MetaboAnalyst platform (https://www.metaboanalyst.ca accessed on 11 May 2026). No sample normalization or data scaling operations were applied to the dataset. To reduce heteroscedasticity and achieve a normal distribution of metabolite intensities, data were subjected solely to a base-10 logarithmic transformation.

Statistical significance across all omics datasets (proteomics, transcriptomics and metabolomics) was assessed using adjusted *p*-values (*p*-adjusted < 0.05), calculated by using a method for multiple testing correction, referred to as FDR in transcriptomic and metabolomic analyses and q-value in proteomic analysis. Additionally, a threshold of log_2_FC ≥ 1 was applied across all omics platforms to identify differentially regulated features.

Principal Component Analyses (PCA) were exclusively employed as an exploratory and descriptive tool to evaluate overall data structure, assess sample clustering, and confirm biological separation between experimental groups.

Functional enrichment analyses for proteome and metabolome data were conducted in the R statistical environment (version 4.5.2) using RStudio (version 2025.09.2+418, Posit Software, PBC, Boston, MA, USA). To identify treatment-induced functional alterations, over-representation analysis (ORA) was performed using the clusterProfiler and ReactomePA packages, mapping differentially expressed molecules against Gene Ontology (GO), Kyoto Encyclopedia of Genes and Genomes (KEGG), and Reactome databases. For transcriptomic data, the Ingenuity Pathway Analysis (IPA) software version 2025 was employed.

To associate the identified differentially expressed molecules with hallmarks of cancer, we employed the consensus framework proposed by Chen et al. (2021) [26].

Omics correlations between transcript and protein levels were quantitatively evaluated using Pearson and Spearman correlation coefficients to determine molecular expression concordance. To interpret the strength of the association, the following criteria were applied: very strong (|ρ|>0.7), moderate (0.7≥|ρ|>0.5), fair (0.5≥|ρ|≥0.3), and poor (|ρ|<0.3). Statistical significance was determined using a threshold of *p* < 0.05.

Protein–protein interaction (PPI) networks for both down-regulated and up-regulated molecules were constructed using the Cytoscape software (version 3.10.4). The interaction data were retrieved from the STRING database (Search Tool for the Retrieval of Interacting Genes; https://string-db.org/).

The clinical value of the selected molecules was determined by constructing Kaplan–Meier survival curves for overall patient survival using the TCGA PanCancer Atlas dataset on HCC (https://www.cbioportal.org/study/summary?id=lihc_tcga_pan_can_atlas_2018 accessed on 15 February 2026).

Finally, for the integration of the different omics layers from unpaired samples, approaches based on the MetaboAnalyst platform (https://www.metaboanalyst.ca accessed on 25 May 2026) and the mixOmics ecosystem were utilized, enabling the identification of coordinated molecular signatures and the visualization of global functional interaction networks modulated by the combination therapy.

## 3. Results

### 3.1. Cellular Proteome Analysis: The Drug Combination Modulated the Expression of Proteins Involved in Hallmarks of Cancer

#### 3.1.1. Identification of Differentially Expressed Proteins in Response to the Drug Combination in HuH7 Cells

**Figure 1:**
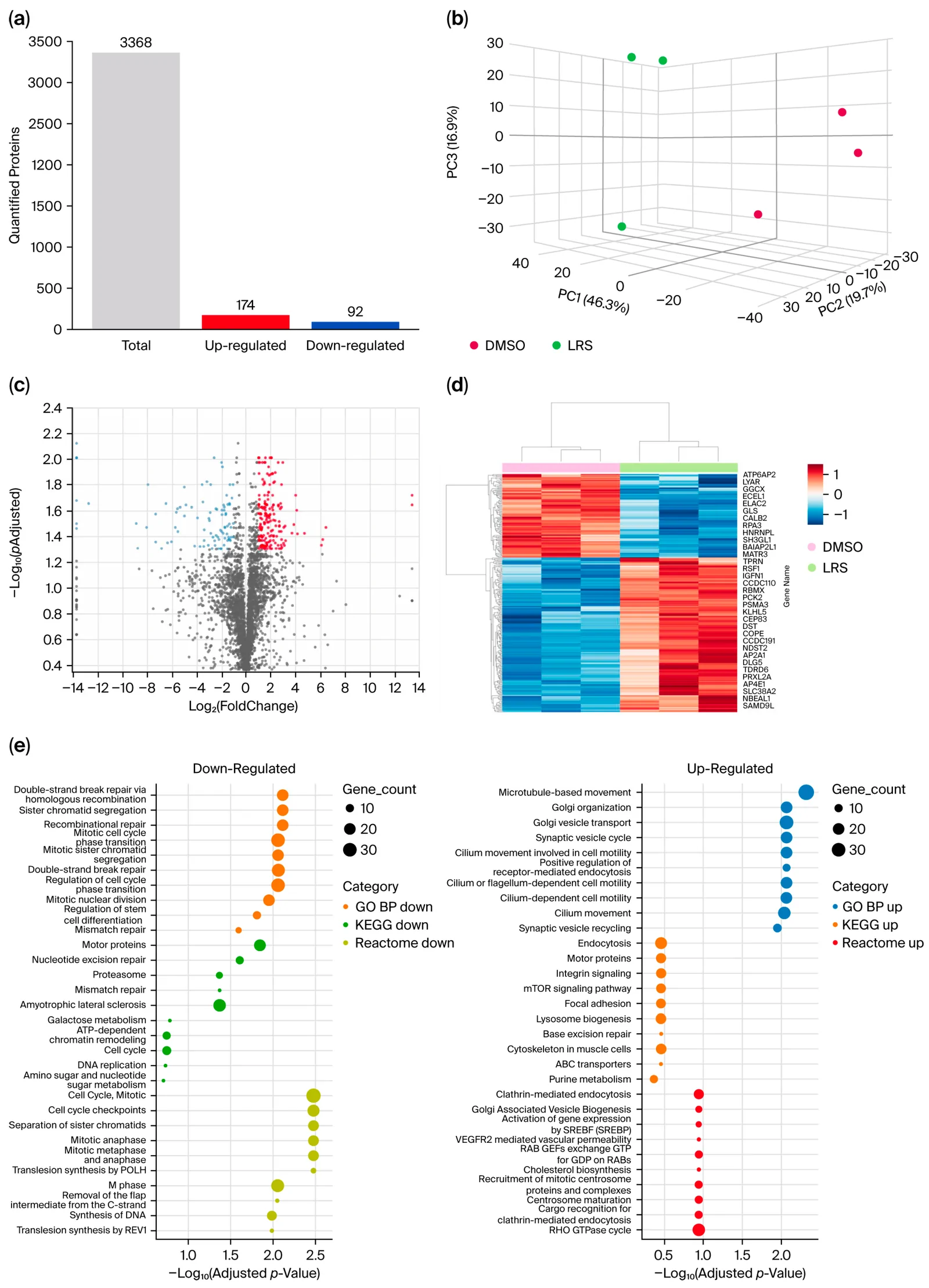
Overall analysis of proteome changes in HuH7 cells treated with the LRS combination. (**a**) Total number of proteins quantified by MS and number of differentially expressed proteins after 72h treatment of cells with the drug combination. (**b**) Principal component analysis (PCA) plot. (**c**) Volcano plot showing differentially expressed proteins. (**d**) Heatmap and hierarchical clustering of protein expression in control samples (DMSO) and samples treated with the LRS combination. Red tones indicate proteins that are up-regulated and blue tones indicate those that are down-regulated in the treatment group compared to the control group. (**e**) Functional enrichment analysis of differentially expressed proteins. For visualization purposes, a cutoff 0.2 was applied.

To assess the overall impact of the drug combination on the proteome of HCC cells, a comparative mass spectrometry analysis was performed on cells treated with the LRS combination versus untreated cells (DMSO-only). A total of 3368 proteins were quantified, of which 266 were differentially expressed after treatment (Figure 1a). Principal component analysis (PCA) revealed a clear separation between the experimental groups (Figure 1b), indicating that the drug treatment induces significant changes in the cell proteomic profile. A total of 82.9% of the total data variability was explained by three principal components. Principal component 1 (PC1) explained 46.33% of the total variability, while PC2 and PC3 explained 19.67% and 16.9%, respectively. The biological replicates for each condition clustered consistently, indicating a marked overall difference between the proteome of the treated cells and the control.

A total of 92 downregulated proteins (Figure 1c, blue dots) and 174 upregulated proteins (Figure 1c, red dots) were identified based on the established selection criteria. Additionally, the heatmap (Figure 1d) shows two predominant expression clusters: one characterized by a decrease in protein levels in treated cells compared to the control, and another showing the opposite tendency. Table 1 lists the top 15 down- and up-regulated proteins, while the complete list is shown in Supplementary Information S1.

Notably, among the differentially expressed proteins, a distinct subset exhibited infinite fold change values (log_2_FC = ±∞), representing exclusive expression patterns. Two groups were identified within this subset: proteins exclusively detected in control samples and completely absent in treatment, suggestive of complete transcriptional silencing or accelerated protein degradation, and proteins undetectable under basal conditions but consistently identified across LRS-treated, indicating condition-specific relative detection upon treatment. To rule out low-intensity missing-value artifacts, we applied strict thresholds requiring binary presence/absence across all biological replicates, statistically significant differential expression (*p*-adjusted < 0.05), and a log_2_FC ≥ 1. This stringent filtering yielded a high-confidence set of 10 ‘infinite’ proteins (Table 2).

Functional enrichment analysis (Figure 1e) revealed that, among the biological processes associated with the down-regulated proteins, DNA repair and the cell cycle are particularly prevalent. Furthermore, the proteins up-regulated following treatment of the cells with the LRS combination are associated with cytoskeletal reorganization and altered lipid metabolism. It should be noted that these enriched terms were identified using a relaxed cutoff of 0.2 for visualization purposes, as the input protein list met the stringent significance criterion of *p*-adjusted < 0.05.

#### 3.1.2. Sets of Differentially Expressed Proteins Associated with Biological Processes Relevant in Carcinogenesis

To gain insights into the potential role of the identified proteins in carcinogenesis, we assessed whether their functions are associated with hallmarks of cancer. We employed the consensus framework proposed by Chen et al. (2021) [26], which establishes the relationship between the hallmarks of cancer and genes using the GO_biological process annotations. Several sets of differentially expressed proteins with annotations in biological processes associated with the hallmarks of cancer were found (Table 3).

#### 3.1.3. Identification of Clinically Relevant Proteins in Patients with Hepatocellular Carcinoma

**Figure 2:**
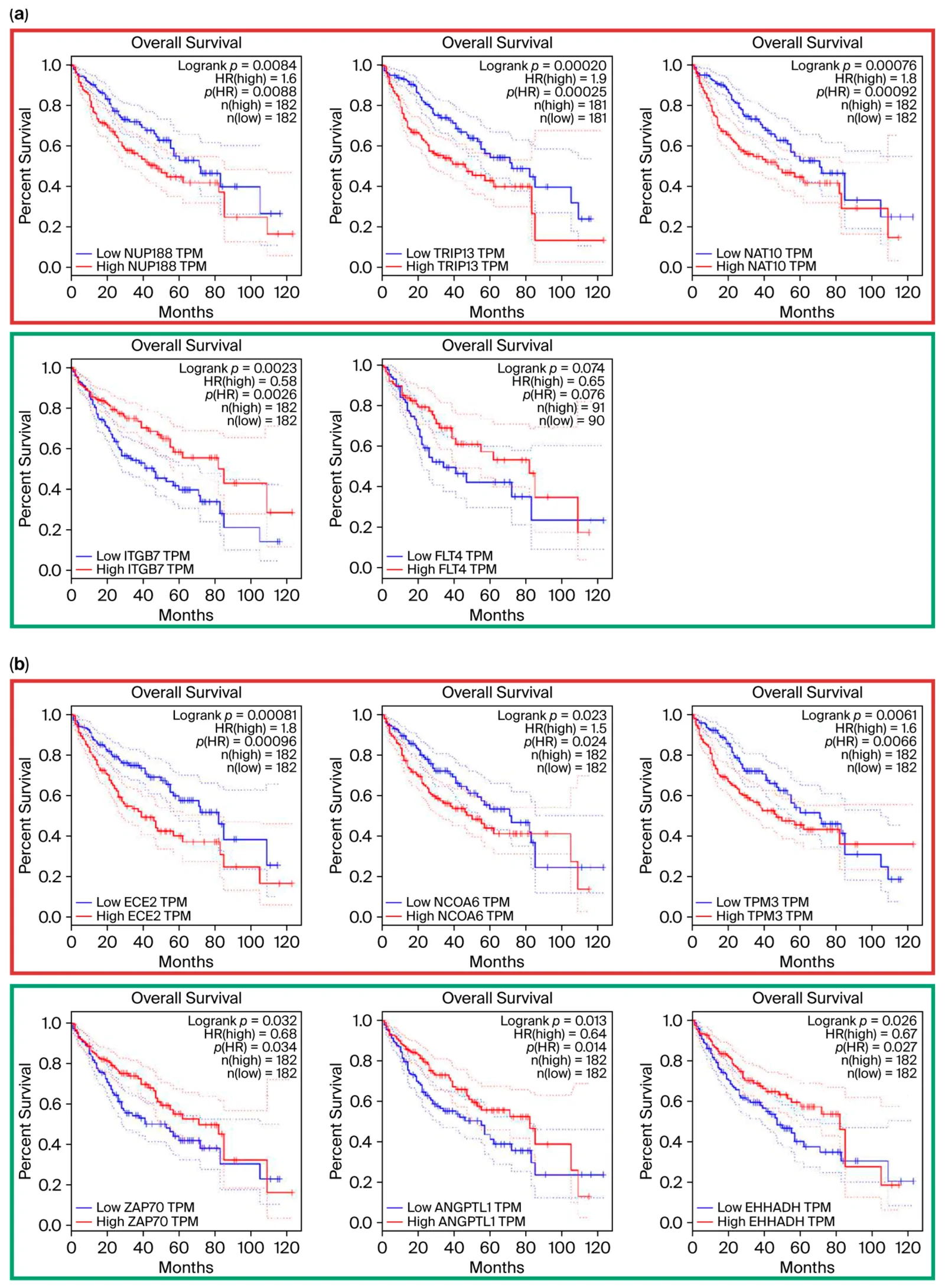
Prognostic clinical value of differentially expressed proteins after drug-combination treatment Kaplan–Meier curves for overall survival in patients with HCC. The graphs show the 10-year overall survival rate associated with mRNA expression levels of differentially expressed proteins after the treatment of HuH7 cells with the LRS combination. Down-regulated (**a**) and up-regulated (**b**) proteins with potential prognostic value. Unfavorable prognosis is shown in the red boxes and favorable prognosis in the green boxes. The dashed lines represent the 95% confidence intervals. Data was obtained from the TGCA-LIHC project.

To determine whether the differentially expressed proteins have clinical relevance in HCC patients, we investigated their association with clinical prognosis in terms of overall survival in the Human Protein Atlas (HPA) database. Table 4 summarizes the total number of proteins associated with favorable or unfavorable clinical prognosis. Analysis of Kaplan–Meier survival curves (from the corresponding mRNA of some differentially expressed proteins) showed that high mRNA expression of genes such as *NUP188* (HR = 1.6(1.09~2.19), *p* = 0.008), *TRIP13* (HR = 1.9(1.37~2.80), *p* = 0.00025), *NAT10* (HR = 1.8(1.33~2.70), *p* = 0.00076), *ECE2* (HR = 1.8(1.27~2.59), *p* = 0.00096), *NCOA6* (HR = 1.5(1.05~2.10), *p* = 0.024) and *TPM3* (HR = 1.6(1.09~2.20), *p* = 0.0061) is significantly associated with poor survival in patients with HCC. On the other hand, high mRNA expression of *ITGB7* (HR = 0.58(0.41~0.83), *p* = 0.0026), *FLT4* (HR = 0.65(0.40~1.06), *p* = 0.076), *ZAP70* (HR = 0.68(0.49~0.98), *p* = 0.032), *ANGPTL1* (HR = 0.64(0.44~0.89), *p* = 0.014) and *EHHADH* (HR = 0.67(0.47~0.95), *p* = 0.026) is associated with better clinical prognosis (Figure 2a,b). These results suggest that the protein expression changes induced by the LRS combination may have clinical relevance in HCC.

### 3.2. Analysis of the Cellular Transcriptome: The Drug Combination Modified the Expression of Genes Involved in the Cell Cycle and DNA Synthesis and Replication

#### 3.2.1. Identification of Differentially Expressed Genes After the Treatment of HuH7 Cells with the Drug Combination

**Figure 3:**
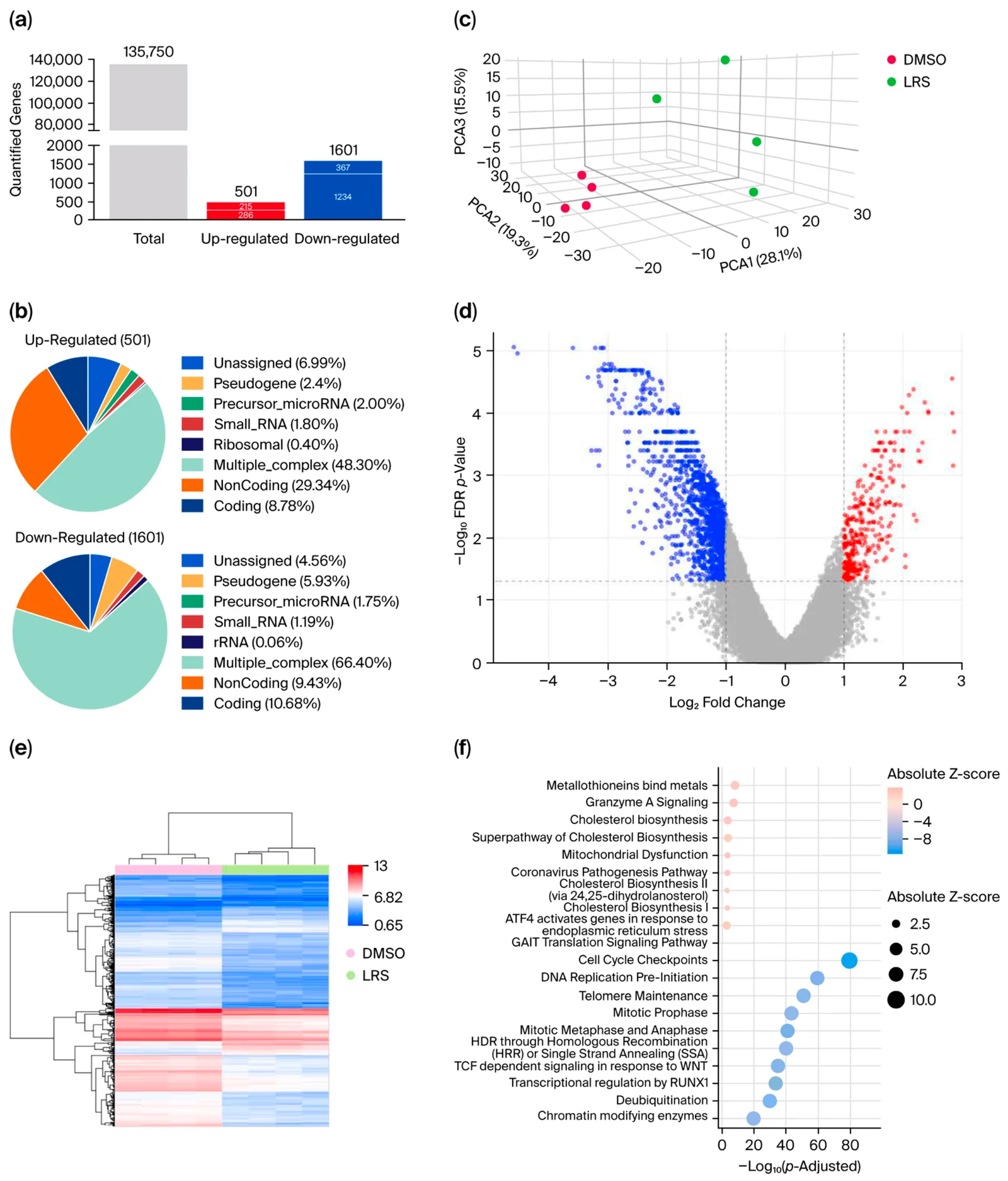
Global analysis of transcriptome changes in HuH7 cells treated with the LRS combination. (**a**) Total number of differentially expressed genes (DEGs) identified in the analysis, showing 2102 genes with significant changes (*p*-adjusted < 0.05 and log_2_FC ≥ 1), of which 1234 were down-regulated (blue) and 286 were up-regulated (red). (**b**) Functional classification of differentially expressed genes according to their transcript type. The pie charts show the distribution of coding genes, non-coding genes, pseudogenes, microRNA precursors, and other categories for both up-regulated (top) and down-regulated (bottom) genes. (**c**) Principal component analysis (PCA) plot. (**d**) Volcano plot representing all identified DEGs. (**e**) Heatmap and hierarchical clustering of gene expression in control samples (DMSO) and samples treated with the LRS combination and (**f**) Top 10 inhibited pathways (negative z-score, blue) and activated pathways (positive z-score, red) with the highest *p*-values in response to treatment with the drug combination. Canonical pathways inferred by the IPA software. For all panels, red represents upregulated genes and blue represents downregulated genes in the treatment group relative to the control group.

To better understand the molecular mechanisms modulated by the LRS combination in HuH7 cells, a transcriptome analysis was performed using cDNA microarrays. Data processing was carried out using ThermoFisher Scientific’s Transcriptome Analysis Console (TAC, version 4.0.1) software. Differential Gene Expression (DEGs) analysis identified 135, 750 genes. Applying a significance threshold of FDR < 0.05 and a fold change of log_2_FC > 1, a total of 2102 genes were considered significantly differentially expressed in response to treatment compared to the control group (Figure 3a,d). Of this total, 1520 were determined to be protein-coding genes (Figure 3a,b).

PCA plot showed the three-dimensional projection of the samples under DMSO (blue) and LRS (red) conditions. A clear separation of the two groups was observed, indicating differential gene expression patterns. The first three principal components (PCA1, PCA2, and PCA3) together explain 63.0% of the total variability in the data, with individual contributions of 28.1%, 19.3%, and 15.5%, respectively (Figure 3c).

Figure 3e shows the heatmap for the control and LRS combination conditions. The heat map reveals a clear separation between the samples treated with the LRS combination and the control samples, suggesting that treatment with the drug combination results in a distinct transcriptomic profile from that of the untreated group. The color scale represents up-regulated genes in red tones, while blue tones represent down-regulated genes relative to the average expression. A distinct set of genes is observed whose expression increases following treatment with LRS, and another group with reduced expression, suggesting a specific transcriptomic modulation induced by this pharmacological treatment. Table 5 shows the *p*-values and fold change values of the main downregulated and upregulated genes in response to treatment. The complete list of DEGs is shown in Supplementary Information S2.

To identify potential molecular processes associated with the transcriptomic changes induced by the drug combination, microarray data were analyzed using Ingenuity Pathway Analysis (IPA) software. This analysis revealed that the main canonical signaling pathways downregulated upon treatment are involved in cell proliferation and cell cycle progression (Figure 3f). Table 6 shows the *p*-values and z-scores for the top 15 inhibited pathways and the top 15 activated pathways in response to treatment. The results were further validated and expanded using functional enrichment analysis (Supplementary Information S3).

#### 3.2.2. Association of DEGs Functions with Biological Processes Relevant in Carcinogenesis

Following the framework consensus strategy proposed by Chen et al. (2021) [26], we identified a group of DEGs with annotations related to biological processes linked to hallmarks of cancer (Table 7). The genes considered for this analysis correspond to those with proven relevance in the cell cycle and DNA synthesis and replication (Table 6). Taken together, these findings suggest that the LRS combination treatment of HuH7 cells led to transcriptomic alterations critical for cell survival. The downregulation of genes involved in cell cycle regulation and DNA maintenance demonstrates that this drug combination effectively shuts down the proliferative machinery characteristic of aggressive HCC. In contrast, the upregulation of stress-responsive pathways suggests that the drugs induce a general cellular stress, ultimately driving cell death.

#### 3.2.3. Identification of Clinically Relevant DEGs in Patients with Hepatocellular Carcinoma

To assess the clinical relevance of DEGs in patients with HCC, we analyzed those whose expression was altered in response to the drug combination and that are involved in key signaling pathways in carcinogenesis, such as the cell cycle, DNA replication, and DNA synthesis. Moreover, a search was conducted in HPA database, revealing that a high percentage of these DEGs are associated with an unfavorable clinical prognosis in patients with HCC (Table 8).

**Figure 4:**
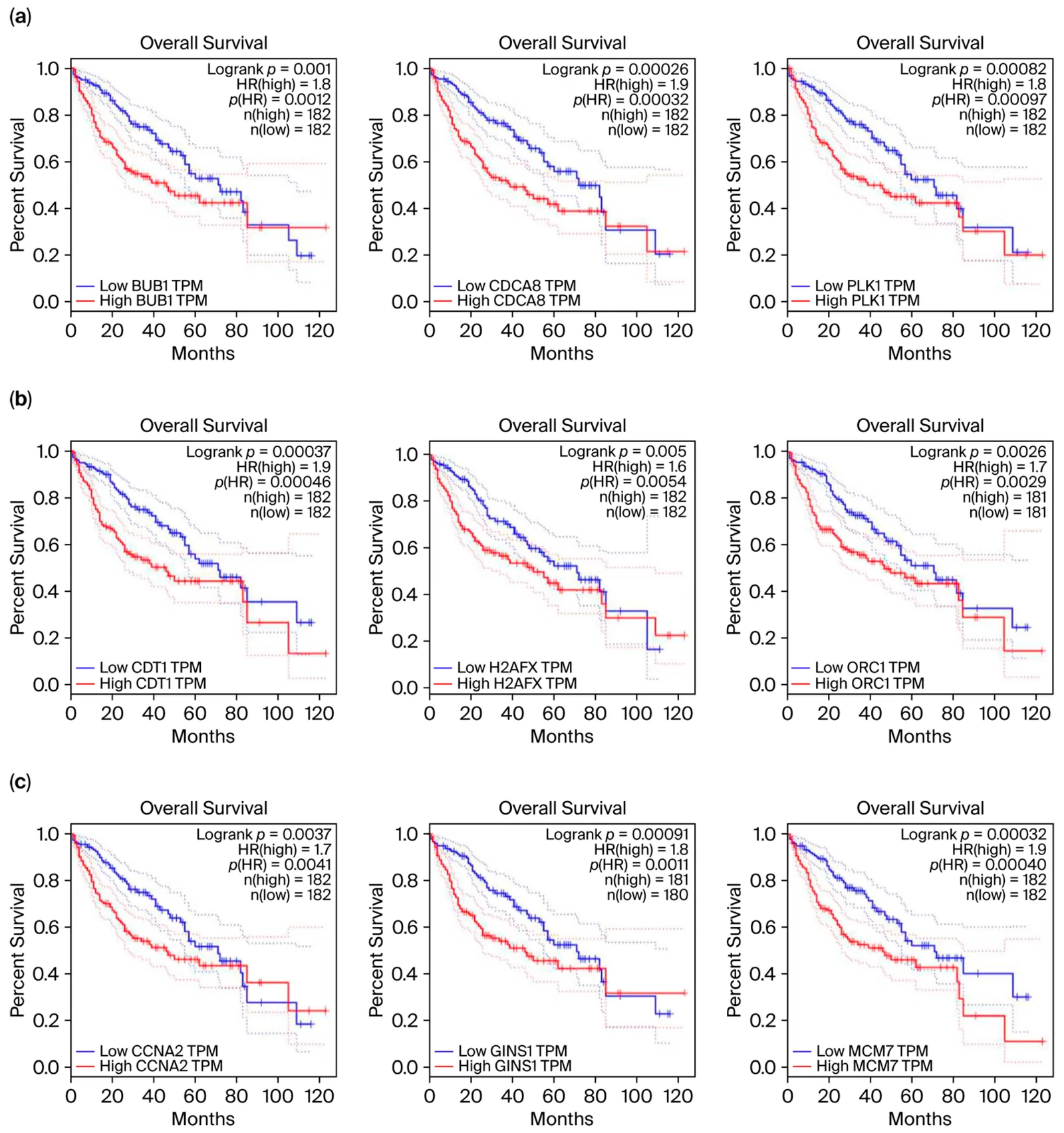
Kaplan–Meier curves for overall survival in patients with HCC. The graphs show the 10-year overall survival rate associated with mRNA expression levels of DEGs in response to treatment with the drug combination. The curves represent genes associated with the signaling pathways: (**a**) cell cycle checkpoints, (**b**) DNA replication, and (**c**) DNA synthesis. Expression of the genes shown is associated with poor clinical prognosis. The dashed lines represent the 95% confidence intervals. Data was obtained from the TCGA-LIHC project.

Analysis of the Kaplan–Meier survival curves revealed that mRNA expression of genes such as *BUB1* (HR = 1.8(1.27~2.57), *p* = 0.001), *CDCA8* (HR = 1.9(1.36~2.77), *p* = 0.00026), *PLK1* (HR = 1.8(1.27~2.59), *p* = 0.00082), *CDT1* (HR = 1.9(1.32~2.69), *p* = 0.00037), *H2AFX* (HR = 1.6(1.13~2.28), *p* = 0.005), *ORC1* (HR = 1.7(1.19~2.41), *p* = 0.0026), *CCNA2* (HR = 1.7(1.19~2.41), *p* = 0.0037), *GINS1* (HR = 1.8(1.27~2.57), *p* = 0.00091) and *MCM7* (HR = 1.9(1.31~2.65), *p* = 0.00032) is associated with poor patient survival (Figure 4). These findings indicate that the LRS combination modulates genes with established prognostic relevance in HCC, supporting the clinical significance of its molecular targets.

### 3.3. Cellular Metabolome Analysis: The Combination of Loratadine, Raloxifene, and Sorafenib Disrupted Key Metabolic Pathways Involved in Cell Survival

#### Identification of Changes in the Abundance of Hydrophilic Metabolites After Treatment of HuH7 Cells with the Drug Combination

**Figure 5:**
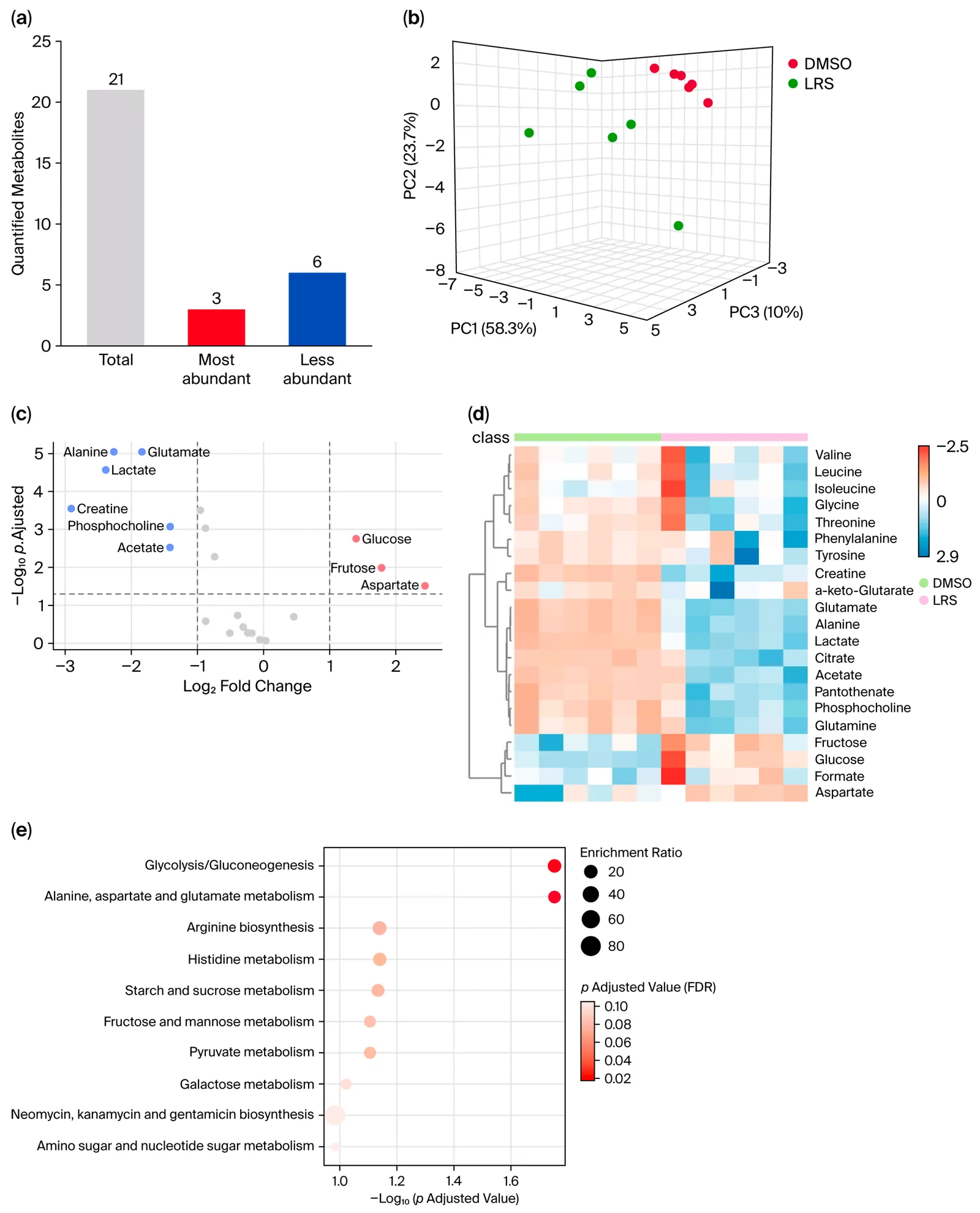
Comprehensive analysis of changes in the metabolome of HuH7 cells treated with the LRS combination. (**a**) Total number of metabolites identified and quantified in the analysis, of which 3 were found to be more abundant and 6 less abundant in the treatment group compared to the control group (log_2_FC ≥ 1 and *p*-adjusted < 0.05). (**b**) Principal component analysis (PCA) plot. (**c**) Volcano plot showing the most and least abundant metabolites (**d**) Heatmap and hierarchical clustering of metabolites in control samples (DMSO) and samples treated with the LRS combination. (**e**) Functional enrichment analysis for metabolites with significant variation in abundance.

To investigate the impact of the LRS combination on the metabolome of HuH7 cells, nuclear magnetic resonance analysis (NMR) was performed 72 h after treatment. 21 metabolites with differential abundance were identified of which 3 showed increased abundance and 6 showed a significant decrease compared to the control group (DMSO, Figure 5a,c). Table 9 shows the list of identified metabolites. The PCA plot shown in Figure 5b reveals a clear separation between the LRS group and the DMSO group, indicating that the LRS combination induced significant changes in the metabolome. The principal components PCA1, PCA2, and PCA3 together explain 92% of the total variability in the data, with individual contributions of 58.3%, 23.7%, and 10%, respectively.

The heatmap shown in Figure 5d also reveals the presence of two clusters among the experimental conditions. In accordance, the drug combination clearly decreased the levels of glutamate, alanine, lactate, creatine, acetate, and phosphocholine, while it increased the levels of fructose, glucose, and aspartate. To determine which metabolic pathways and/or biological processes are associated with the altered metabolites following treatment, a functional enrichment analysis was performed (Figure 5e). This analysis significantly showed that prominent modulated processes include the glucose metabolism and alanine, aspartate and glutamate metabolism.

### 3.4. Integrative Proteomic–Transcriptomic-Metabolomic Analysis Reveals Key Molecular Networks Involved in Carcinogenesis

#### Integrative Proteomic–Transcriptomic-Metabolomic Analysis

**Figure 6:**
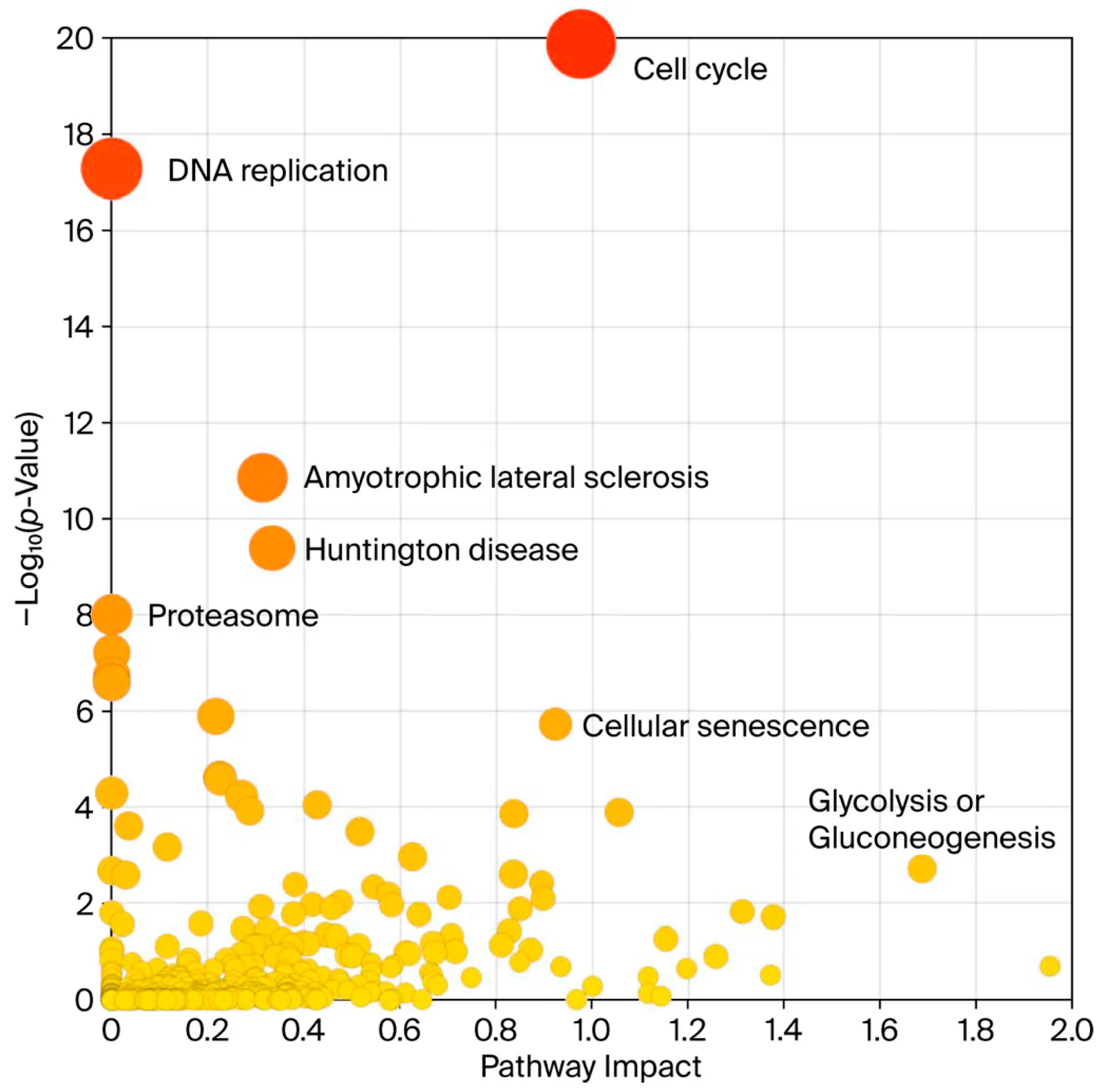
Joint pathway enrichment analysis. The joint pathway enrichment plot generated using MetaboAnalyst shows the altered proteins, transcripts, and metabolites after HuH7 cells treatment with the LRS combination compared to the control group. The size and color of the points are proportional to the enrichment level (number of associated molecules) and statistical significance, respectively. Pathways with the highest statistical significance are shown in red.

To identify the signaling pathways globally altered by the LRS combination, quantitative profiles of transcripts, proteins, and metabolites were integrated into a joint pathway analysis performed using MetaboAnalyst (Figure 6). This analysis revealed main altered pathways in HuH7 cells and those most representative of carcinogenesis were DNA replication, the cell cycle, proteasome activity, and cellular senescence.

The integrated data in this analysis provides strong evidence that combination therapy targets multiple pathways in HCC, synergistically disrupting essential mechanisms for cell survival.

Because mRNA levels do not always directly correlate with functional protein abundance due to post-transcriptional regulation and protein stability, we next performed an integrative analysis of these two datasets. To evaluate the relationship between the two “omics”, we employed the following approaches:

(a) Global Analysis: We evaluated the matrix of all molecules detected in both datasets without prior significance filtering. These results are detailed in Supplementary Information S4 and show a correlation involving 3040 genes. The Spearman correlation was higher than the Pearson correlation (0.132 vs. 0.070), confirming that the relationship between the transcriptome and proteome is non-linear influenced by outlier or non-proportional ranges. The significance value between 2.753×^−13^–1.073×^−14^ is of high statistical significance although there is very low overall correlation (r ≈ 0.0702–0.1320); this means the trend (albeit weak) is genuine and not a random artifact.
(b) Unidirectional Analysis: We applied the significance filters (log_2_FC ≥ 1 and *p*-adjusted < 0.05) to only one “omic” at a time, measuring the behavior of the other dimension in an unconstrained, independent manner. This analysis revealed two distinct response patterns to the treatment. First, genes showing significant transcriptional alterations following treatment (log_2_FC ≥ 1 and *p*-adjusted < 0.05) partially transmitted this signal to the proteome (r = 0.165, *p* = 0.00225). This demonstrates that a fraction of the cellular response to the LRS combination is indeed mediated by classical transcription-translation axis; second, proteins whose concentrations were drastically altered by the treatment did not show a significant correlation with their respective mRNA levels (r = 0.118, *p* = 0.067). These data are shown in Supplementary Information S4.

**Figure 7:**
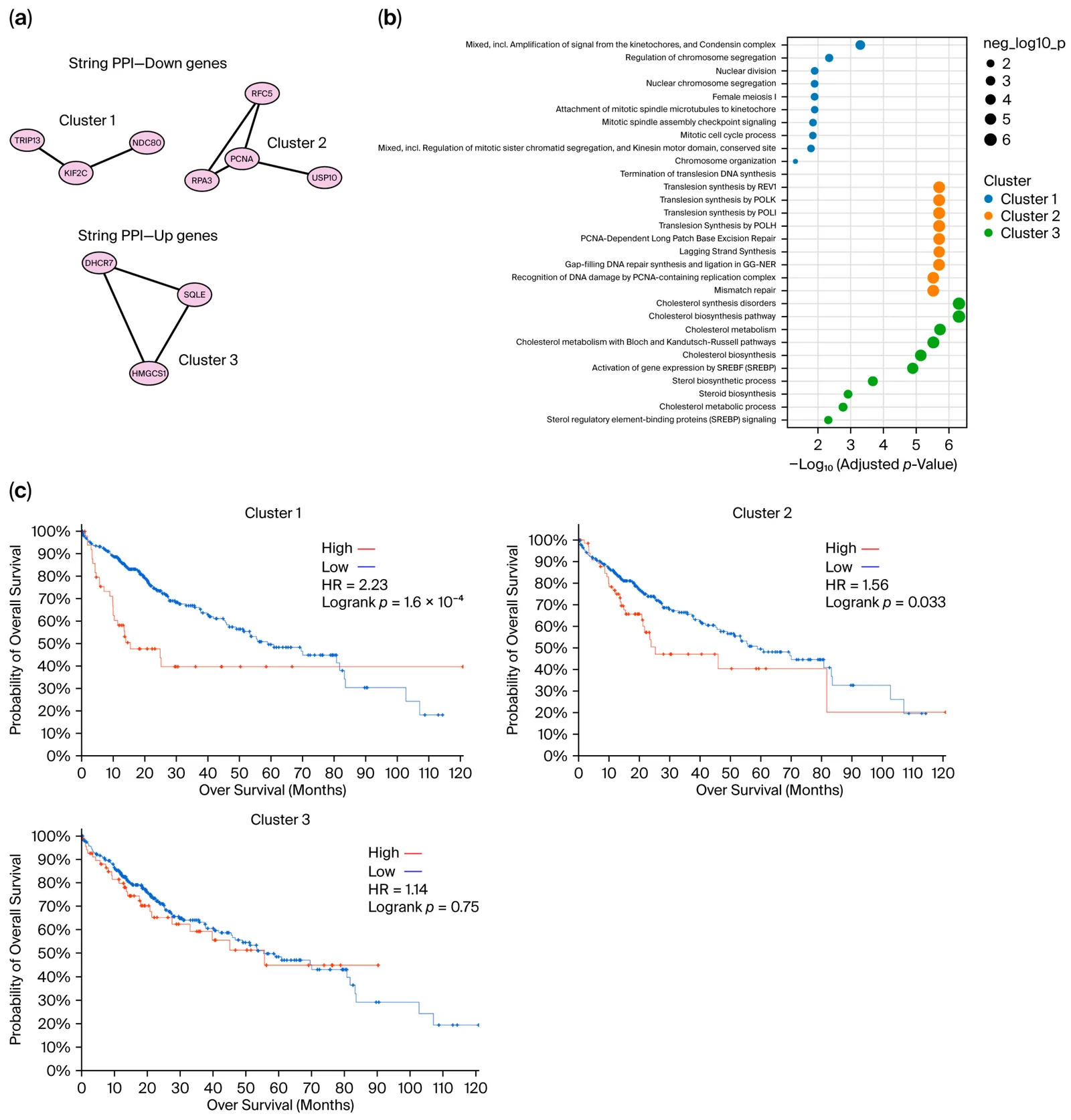
The transcriptomic-proteomic profile after treatment of HuH7 cells with the LRS combination. Integration of proteomic and transcriptomic datasets reveals concordant gene regulation and associated biological processes. (**a**) PPI networks derived from STRING for downregulated and upregulated genes, grouped into functional clusters (Louvain algorithm). (**b**) Top functional enrichment pathways for each network cluster (*p*-adjusted < 0.05). (**c**) Kaplan–Meier curves evaluating overall survival (OS) probability in the TCGA-LIHC cohort stratified by High (red line) versus Low (blue line) composite expression score of Cluster 1, 2 and 3 genes.

We next constructed composite module scores based on the Louvain-detected clusters from our STRING hub network. We found 12 hubs (number of upregulated clusters detected = 1, number of downregulated clusters detected = 2) (Figure 7a). The network of down-regulated molecules revealed highly interconnected critical nodes such as *PCNA* and *RPA3*, whose enrichment was enhanced in processes associated with the cell cycle and DNA replication (Figure 7b and Table 10). On the other hand, the up-regulated molecules formed a smaller network consisting of *DHCR7*, *SQLE*, and *HMGCS1*, which showed significant enrichment in processes related to cholesterol synthesis, steroid metabolism, and SREBP-mediated gene activation (Figure 7b and Table 10).

Figure 7c and Table 11 shows the Kaplan–Meier and log-rank analyses based on median cluster scores, which demonstrated that high baseline expression of specific functional modules (Cluster 1 and Cluster 2) significantly stratifies patient overall survival (HR = 2.23 (1.26–3.95), *p* = 0.00016 and HR = 1.56 (0.97–2.5), *p* = 0.033, respectively).

To determine whether the prognostic value of cluster 1 is specific or merely reflects a generic cell proliferation response, we performed a multivariable Cox proportional hazard regression analysis in the LIHC-TGCA cohort (n = 365). The model simultaneously incorporated the composite score of Cluster 1, Cluster 2 (which contains the canonical proliferation marker *PCNA*), and individual *PCNA* mRNA expression as canonical proliferation control. Remarkably, Cluster 1 remained a statistically independent predictor of survival (HR = 1.86, 95% CI = 1.40–2.47, *p* = 0.001), whereas neither cluster 2 (HR = 0.8, 95% CI = 0.55–1.23, *p* = 0.33) nor individual *PCNA* (HR = 0.96, 95% CI = 0.70–1.32, *p* = 0.81) retained prognostic significance when mutually adjusted (Supplementary Information S5).

**Figure 8:**
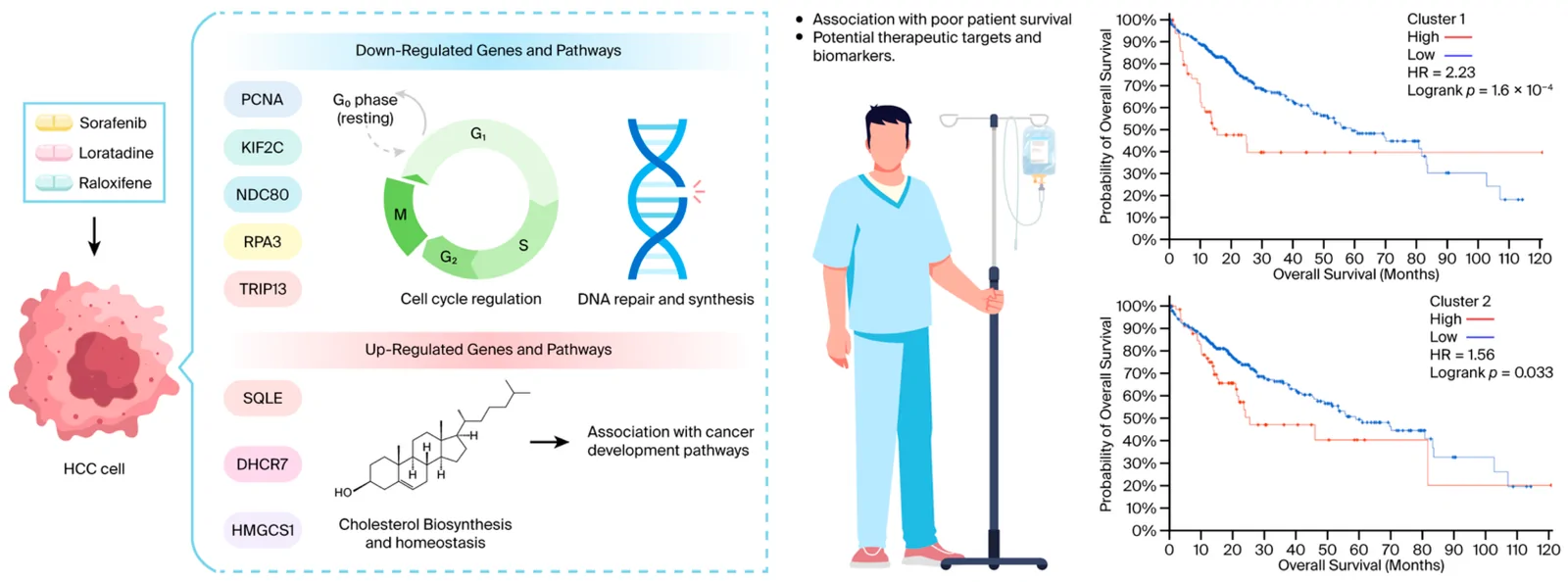
Proposed proteomic-transcriptomic integrative anticancer mechanism of the LRS combination. The schemes illustrate how concordant proteomic and transcriptomic alterations induced by LRS treatment converge on key regulators of the mitotic cell cycle, DNA repair and synthesis, and cholesterol metabolism. Key nodes relevant to these biological processes were identified; those down-regulated in response to treatment were associated with the cell cycle and DNA repair, while up-regulated molecules were linked to cholesterol biosynthesis. Their coordinated dysregulation is associated with impaired mitotic progression, ultimately leading to tumor cell death. Kaplan–Meier curves for overall survival in HCC patients (from the TCGA-LIHC project) indicate that the expression levels of some of these molecules are associated with prognosis. This clinical association further supports the potential prognostic and therapeutic value of these targets.

In summary, the integrated results provided a basis for proposing a mechanistic model in which the concordant proteomic and transcriptomic alterations induced by the LRS combination converge on regulators of DNA repair (Figure 8). Based on this analysis, we identified down-regulated genes whose activity is associated with cell mitosis and DNA repair and synthesis (*PCNA*, *KIF2C*, *NDC80*, *RPA3* and *TRIP13*) and up-regulated genes involved in cholesterol biosynthesis (*SQLE*, *DHCR7* and *HMGCS1*). According to our data, the LRS combination in HuH7 cells decreased the expression of these molecules at both the RNA and protein levels, suggesting that, collectively, loratadine, raloxifene, and sorafenib may be downregulating the cell cycle, thereby promoting the induction of cell death.

Interestingly, analyses of the Kaplan–Meier curves from the TCGA-LIHC project show that high expression of these genes is associated with poorer overall survival in patients, supporting their relevance as potential prognostic biomarkers and therapeutic targets (Figure 8). Taken together, these findings suggest that modulation of these central nodes could be a key mechanism underlying the antitumor effect of the LRS combinations on HCC cells.

To evaluate the relevance of the representative hub (*PCNA*, *KIF2C*, *NDC80*, *RPA3* and *TRIP13*), we investigated their baseline expression profiles and functional dependency across liver cancer cell lines panels (from CCLE and DepMap datasets) and data from patients (from LIHC-TCGA project).

At the transcriptomic level, analysis of baseline RNA sequencing data (log_2_ (TPM+1), CCLE Public 26Q1) demonstrates basal expression of all five genes across HCC cell lines (Supplementary Information S6). Moreover, protein abundance was assessed using quantitative mass spectrometry (z-scores, Harmonized MS CCLE Gygi dataset). All target proteins displayed detectable baseline expression in selected HCC cells (Supplementary Information S7).

Finally, to establish the functional requirement of these components for cell survival and growth, we investigated the genomic dependency scores derived from genome -scale CRISPR-Cas9 knockout screens (Chronos dependency score, DepMap Public 26Q1). Knockout of each individual gene resulted in marker negative Chronos scores (≤−0.5) across several HCC cell lines, including HuH7 cells (Supplementary Information S8).

At clinical level, the cohort data from LIHC-TCGA project shows that this target gene cluster is altered across HCC samples. It is essential to emphasize that these alterations are primarily driven by mRNA overexpression and amplification (Supplementary Information S9). This profile of clinical alterations highlights that the overexpression of this group of genes constitutes a clinically relevant feature in a subgroup of patients with HCC, supporting its potential role as a biomarker of proliferative aggressiveness and as a therapeutic target.

Taking together, these findings suggest that *PCNA*, *KIF2C*, *NDC80*, *RPA3* and *TRIP13* are actively expressed at both mRNA and protein levels, representing functional vulnerabilities in HuH7 and other cellular models of HCC.

## 4. Discussion

HCC is characterized by marked molecular heterogeneity, which contributes to therapeutic resistance and limited treatment efficacy. Despite advances in targeted therapies, current treatment options remain limited and offer poor clinical benefit. Although the cytotoxic effects of LRS treatment were previously documented by our research group [19], the molecular mechanisms underlying this response remained unclear. Here, the integrative analysis of proteome, transcriptome, and metabolome profiles provides, for the first time, a systematic target-prioritization framework, revealing potential mechanisms underlying the cytotoxic effects of the LRS combination in HuH7 cells.

Quantitative characterization of the proteomic profile of cells exposed to the combination revealed 174 upregulated proteins; functional enrichment analysis indicated that the associated biological processes were cell motility, the Hippo pathway, cholesterol biosynthesis, and the GTPase cycle. On the other hand, 92 proteins displayed decreased expression following treatment with the triple combination and were associated with enriched biological processes including cell cycle regulation (specifically mitosis), cytoskeletal organization, and DNA synthesis and repair.

A remarkable finding in the proteomic analysis was the identification of proteins with a binary expression pattern, a switch-like nature of their expression, which may reflect irreversible cellular reprogramming rather than a graded transcriptional response, characterized by their detection only in the control group and their complete absence in the treatment group, or vice versa (proteins with Infinity score, Table 2). The integration with the matched transcriptomic data revealed two distinct biological mechanisms: (a) transcriptional silencing (50% n = 5), half of these targets displayed corresponding negative mRNA shifts under treatment, confirming aligned downregulation; (b) post-translational clearance (50%, n = 5), the remaining targets maintained stable mRNA expression despite complete protein loss, pointing to treatment-induced post-translational degradation rather than technical detection failure (Supplementary Information S10). Regarding this, preclinical studies have identified several key drivers of hepatocarcinogenesis, including C7orf50 (promoter of progression and metastasis via NF-κB/PAI-1) [27], *NUP188* (essential for stemness and pluripotency) [28], and *HS2ST1* (promoter of cell cycle and mTORC1 signaling) [29], all of which correlate with poor clinical prognosis. Notably, treatment with the LRS combination completely suppressed these oncogenic proteins. Furthermore, multi-omics integration suggests that LRS-mediated silencing of *NUP188* impairs nuclear transport, leading to cytoplasmic protein accumulation, endoplasmic reticulum stress, and activation of the proteasome and ALS-related stress pathways.

The transcriptomic profile of HuH7 cells indicated that, after incubation with the LRS combination, the expression of 1601 transcripts decreased, of which 1234 were coding transcripts; in addition, an increase in the expression of 501 transcripts was observed, 286 of which were from the coding group (Figure 2a). Overall, functional enrichment analysis showed that, among the downregulated processes, the cell cycle and DNA regulation were once again the most prominent; conversely, the upregulated processes were those related to granzyme A and metallothionein signaling and cholesterol biosynthesis (Figure 3f and Table 6). In this context, some studies have linked granzyme activity to antitumor response and the induction of pyroptosis in various cancers [30]; on the other hand, metallothioneins are downregulated in HCC, and specifically MTG1 acts as a tumor suppressor in this cancer [31,32].

Analysis of changes in the cellular metabolome revealed significant alterations in the abundance of 9 metabolites; specifically, the LRS combination reduced the abundance of alanine, glutamate, lactate, creatine, acetate and phosphocholine; while increasing the abundance of glucose, fructose and aspartate.

The metabolic networks of cancer cells are altered in order to promote their malignant characteristics; therefore, metabolic reprogramming is a key feature in the development and progression of the disease [33,34]. Compared to normal cells, cancer cells require a higher source of biofuels. Thus, glutamate is an essential component for their survival and proliferation [35,36]. Lactate, in addition to serving as an energy source, is a substrate for lactylation, a post-translational modification that plays a significant role in tumor proliferation, the regulation of immune cell function, and the modulation of gene expression [37]; in fact, Cheng and colleagues propose lactate and lactylation as promising novel targets for the treatment of liver diseases. In colorectal cancer, phosphocholine levels are elevated, and it is suggested that it plays an important role, along with choline kinase (an enzyme that catalyzes the phosphorylation of choline to form phosphocholine), in inducing cell proliferation and signal transduction during carcinogenesis [38]. Acetate, in addition to being a substrate for the synthesis of acetyl-CoA that enters the Krebs cycle, is an essential component in epigenetic regulation [39]. The significant decrease in the abundance of these metabolites suggests that the LRS combination antitumor effects may be taking place by disrupting the main metabolic pillars of HuH7 cells, at least partially.

On the other hand, treatment with the LRS combination induced a significant increase in the intracellular abundance of glucose, fructose, and aspartate in HuH7 cells. Although cancer cells typically accelerate glucose uptake to use it for lactate production regardless of the presence of oxygen, (the very well-known Warburg effect that supports cell survival and proliferation [33,34,40]), the increase in glucose observed in this study suggests a severe metabolic blockade that inhibits its utilization. This disruption in glycolytic flux is supported by the significant decrease in lactate levels also reported in this study, which points to an energy and biosynthetic collapse of the tumor. Similarly, the intracellular accumulation of fructose exacerbates this metabolic paralysis; although this monosaccharide is crucial in the development of HCC by enhancing mitochondrial activity, ATP production, and angiogenesis [41]. Our results suggest that the treatment with the drug combination inhibits catabolism, leading to its intracellular accumulation. Finally, this metabolic dysregulation also involves aspartate, an amino acid identified as the absolute limiting factor for tumor replication under conditions of metabolic stress and hypoxia, where its availability modulates the synthesis of essential macromolecules and the function of the electron transport chain [42]. Consequently, the simultaneous accumulation of glucose, fructose, and aspartate suggests that the LRS combination systemically disrupts the consumption of HCC most critical energy and structural substrates, thereby disabling its proliferative capacity.

The multiomic integration led to the identification of five key molecules (*KI2FC*, *PCNA*, *TRIP13*, *NDC80*, and *RPA3*) that play a critical role in cell cycle regulation, DNA homeostasis, and cholesterol metabolism (Figure 8). Furthermore, the integrative analysis of the three omics profiles confirmed that the LRS combination modulates key components of the cell cycle and DNA replication (Figure 6). The global analysis (n = 3040) revealed a weak coupling between the transcriptome and the proteome (Spearman r = 0.1320, Supplementary Information S4), demonstrating that RNA abundance does not directly determine protein levels in the system. When evaluating the response to treatment impartially via unidirectional analyses, it was observed that genes with an active transcriptional response (n = 360, significant RNA only) transmit a moderate yet significant signal to their corresponding proteins (r = 0.1649, *p* = 0.0022). Conversely, in the subset of proteins showing drastic changes (n = 241, significant protein only), the correlation with their corresponding RNAs disappears (r = 0.1181, *p* = 0.0671). Taken together, this asymmetry demonstrates that the treatment-induced cellular reprogramming is not executed via the classical transcription-translation axis, but rather through a predominance of post-translational regulatory mechanisms (such as changes in protein stability, degradation, or translation rate).

Although our transcriptomic data are consistent with the findings reported by Contreras et al. [43] regarding the inhibition of DNA replication and the cell cycle as a conserved effect of sorafenib, the LRS combination suggests significant advantages over monotherapy. In contrast to the responses induced by this multikinase inhibitor alone—such as specific inhibition of the cell cycle, preferential alteration of lipid metabolism [44], or the activation of adaptive protein responses for survival and drug resistance [45]—the LRS combination achieves a greater multi-omic impact using a lower concentration of the base drug (4.04 µM). Specifically, the LRS combination dismantles tumor plasticity by triggering robust transcriptomic modulation and a comprehensive bioenergetic collapse. It also drastically disrupts genomic integrity and the cell division machinery, highlighting the presence of a group of 10 proteins exhibiting a binary pattern (log_2_FC = ±∞). This binary change pattern suggests that the addition of loratadine and raloxifene could contribute to the modulation of key survival nodes that are not completely inactivated by sorafenib monotherapy.

To link our experimental findings to clinical practice, we analyzed patients’ stratification based on expression clusters of the targeted signaling network. Importantly, patients characterized by high co-expression of these molecular nodes exhibited significantly reduced survival (Figure 7c). However, it is important to acknowledge that this K-M analyses are based on mRNA expression data from TCGA project. While transcriptomic data provides extensive clinical and prognostic information, mRNA abundance does not always directly reflect functional protein levels or post-translational activation. Thus, future studies should focus on incorporating protein expression data to validate such clinical findings.

A complete analysis of the biological implications of the nodes modulated by the three- drug combination is deserved. Nevertheless, we describe briefly five of the modulated nodes to gain some insights into the possible specific antiproliferative molecular mechanisms of the LRS combination in HCC cells and their clinical association. (1) Kinesin family member 2C (*KIF2C*) regulates microtubule dynamics during mitosis and is closely linked to various hallmarks of cancer [46], chemoresistance [47,48] and poor clinical prognosis in HCC [49,50,51]. Mechanistically, *KIF2C* upregulation via the Wnt/β-catenin pathway drives proliferation, migration, and metastasis in HCC [50], whereas its silencing increases cisplatin sensitivity through PI3K/AKT/MAPK signaling [52]. (2) Proliferating cell nuclear antigen (*PCNA*) is a key regulator of cell proliferation, DNA replication, and repair, serving as an established clinical biomarker in oncology [53]. In HCC, *PCNA* is significantly upregulated in malignant compared to benign tissue, directly correlating with tumor aggressiveness, cell cycle activation, and poor overall survival [54,55,56]. Mechanistically, targeting *PCNA* pathway dynamics—such as inducing its ubiquitination via USP1 inhibition—sensitizes HCC cells to cytotoxic chemotherapy [57]. (3) Thyroid hormone receptor interactor 13 (*TRIP13*) is an AAA+ ATPase that regulates spindle assembly checkpoints, DNA repair, and tumorigenesis across multiple cancers [58,59,60]. In HCC, *TRIP13* drives progression by modulating the AKT/mTOR pathway through its interaction with *ACTN4* [61], while its silencing or therapeutic inhibition significantly impairs proliferation, migration, and invasion [62,63,64]. (4) Kinetochore complex component (*NDC80*) is widely overexpressed across cancers and correlates with poor prognosis [65]. In HCC, *NDC80* is a key biomarker in aggressive subtypes and HBV-driven carcinogenesis, promoting tumor growth, migration, and hypoxia-induced resistance to anti-PD-L1 immunotherapy [66,67,68]. Clinically, *NDC80* knockdown impairs proliferation, induces cell cycle arrest and apoptosis [69], and exhibits high binding affinity to first-line tyrosine kinase inhibitors like sorafenib, lenvatinib, and regorafenib [67,70]. (5) Replication protein A3 (*RPA3*) is a subunit of the heterotrimeric RPA complex involved in DNA metabolism and oncogenesis [71]. Although understudied in liver cancer, *RPA3* is significantly elevated in HCC tissue and correlates with increased cell proliferation, tumor progression, poor clinical prognosis, and radiotherapy resistance [72]. Notably, suppressing *RPA3*—such as via miR-146a-5p targeting—enhances therapeutic sensitivity in HCC cells [72].

These findings suggest that the LRS combination negatively modulates a network of genes essential for cancer cells, inducing irreversible cellular and metabolic collapse.

Finally, the integrative analysis showed that exposing HuH7 cells to the LRS combination significantly increased cholesterol biosynthesis. This increase appears to be a survival response by the cells to stress induced by the drug. Interestingly, Huang et al. argue that tumor cells have a higher demand for cholesterol to maintain their membrane structure and ensure proliferation signals. When faced with stressful conditions, it is common for lipid pathways to be reprogrammed as a defense mechanism. At the molecular level, this mechanism is activated via the mevalonate pathway, where the transcription factor *SREBP2* translocates to the nucleus to trigger the expression of key target enzymes such as *HMGCR* and *SQLE* [73]. In fact, the role of *SQLE* and other enzymes such as ACAT1 has already been linked to the progression and aggressiveness of HCC [74]. Genomic data from the TCGA consortium reveal that *SQLE* is a gene that is significantly overexpressed in liver tumors and that it promotes cell growth by activating pathways such as ERK and Akt [75]. Crucially, this defensive response lays bare the tumor’s evasion strategy, presenting an opportunity to design a combination therapy that deliberately disrupts the metabolic adaptation driven by the LRS combination.

Our integrative multi-omics approach demonstrates for the first time that the LRS combination potently and simultaneously inhibits nodes essential for tumor development, progression, and drug resistance in HCC. While our study provides a comprehensive multi-omics integration, we acknowledge that these findings represent a proof-of-concept discovery in HuH7 cells. A primary limitation of the present work is the absence of direct functional or molecular validation, such as qRT-PCR, Western blotting, or cell viability and apoptosis assays, to confirm the predicted molecular mechanisms. Thus, the mechanisms proposed herein should be interpreted as candidate hypotheses derived from integrated multi-omics analysis. Future in vitro and in vivo studies will be essential to validate these priority targets and elucidate their precise molecular execution for potential clinical translation. These findings expand upon what has been previously reported about the relevance of such nodes. Thus, this combination represents a robust therapeutic strategy capable of modulating key molecular targets in hepatic carcinogenesis, providing a scientifically significant basis with major clinical relevance that supports its use as a promising therapeutic alternative.

## 5. Conclusions

In summary, this study provides a proof-of-concept discovery in HuH7 cells, highlighting the efficacy of multi-omics integration to unveil key molecular targets and pathways modulated by the LRS combination. These findings provide valuable candidate biomarkers and potential therapeutic targets for HCC, laying the groundwork for future research. Multi-omic integration suggests that the combination drug therapy completely suppresses critical oncogenic drivers (*C7orf50*, *NUP188*, and *HS2ST1*) and downregulates central nodes (*KIF2C*, *PCNA*, *TRIP13*, *NDC80*, and *RPA3*) directly associated with a poor clinical prognosis in patients with HCC. Additionally, our findings indicate that the treatment induces a bioenergetic collapse by modulating the metabolism of molecules critical for cell survival.

The synchrony in down- or up-regulation of several molecules, as well as its association with clinical prognosis in HCC patients and the correlation with carcinogenesis pathways and processes, highlight the relevance of the findings. Nevertheless, future perspectives should focus on functional validation to confirm their individual and collective contributions to the observed cytotoxic phenotype. Utilizing CRISPR-Cas9 gene editing or siRNA-mediated knockdown assays in parallel with LRS treatment will clarify whether the suppression of these specific hub nodes is the primary driver of the cytotoxic effect, or if they act as independent parallel targets. However, a key limitation in public repositories such as the TCGA dataset is the lack of standardized longitudinal drug-treatment annotations. Consequently, while our findings establish this composite hub score as a robust prognostic biomarker for patient risk stratification, its prospective utility as a predictive signature for treatment response to targeted agents warrants further validation in prospectively annotated clinical trial cohorts. Moreover, in the context of personalized medicine, future clinical trials should investigate this regulatory signature as a predictive stratification tool; patients presenting with baseline overexpression of these aggressive hub molecules could be selected as ideal candidates who would derive the highest therapeutic benefit from the LRS combination.

To our knowledge, this is the first report of the effect of loratadine and raloxifene at the omics level. This multi-omics approach unveiling cancer cell network rewiring may help in the discovery of several anticancer targets as well as in revealing the participation of diverse molecules (Table 1, Table 2, Table 5 and Table 10, and Supplementary Information S1–S10) in cancer progression and prognosis not only in HCC but in a plethora of cancers and diseases.

## Figures and Tables

**Table 1:** List of the main differentially expressed proteins after the treatment with the LRS combination.

**Downregulated**					
**Uniprot Accession**	**Name**	**Avg (log_2_)**		**Log_2_FC**	***p*** **Adjusted **
		**LRS**	**DMSO**		
Q6ZN84	Coiled-coil domain-containing protein 81	0.387	13.103	−12.72	0.022
Q8IV04	Carabin	2.683	11.562	−8.88	0.032
A0A1B0GTU8	Renin receptor	1.212	9.970	−8.76	0.049
G3V5E8	Galactocerebrosidase	1.799	9.840	−8.04	0.034
C9J2C7	NT5C1B-RDH14 readthrough	−0.712	7.218	−7.93	0.016
Q9ULE0	Protein WWC3	1.930	9.234	−7.30	0.027
P02647	Apolipoprotein A-I	2.974	10.274	−7.30	0.044
Q8IYJ1	Copine-9	3.916	10.747	−6.83	0.049
P22676	Calretinin	2.796	9.274	−6.48	0.022
A0A8Q3SIY9	Actin filament associated protein 1 like 2	2.456	8.812	−6.36	0.045
Q9UKN7	Unconventional myosin-XV	0.392	6.424	−6.03	0.044
Q96DI7	U5 small nuclear ribonucleoprotein 40 kDa protein	4.196	9.921	−5.73	0.034
H0Y8U5	C-terminal binding protein 1	5.145	10.794	−5.65	0.026
Q05682	Caldesmon	5.311	10.870	−5.56	0.024
B4DUC8	S-methyl-5′-thioadenosine phosphorylase	6.258	11.779	−5.52	0.036
**Upregulated**					
**Uniprot Accession**	**Name**	**Avg (log_2_)**		**Log_2_FD**	***p*** **Adjusted**
		**LRS**	**DMSO**		
Q96PQ7	Kelch-like protein 5	7.796	1.351	6.45	0.034
Q14588	Zinc finger protein 234	12.835	6.447	6.19	0.042
Q9UPM8	AP-4 complex subunit epsilon-1	12.271	6.188	6.08	0.047
P0DPD6-3	Isoform ECE2-3 of Endothelin-converting enzyme 2	11.059	6.346	4.71	0.038
A0A669KB88	P-type Cu (+) transporter	9.188	5.037	4.15	0.038
F6M2K2	Nuclear receptor coactivator 6	10.699	6.607	4.09	0.025
O00370	LINE-1 retrotransposable element ORF2 protein	11.305	7.295	4.01	0.019
Q8NCU4	Coiled-coil domain-containing protein 191	12.441	8.579	3.86	0.032
P06753	Tropomyosin alpha-3 chain	16.130	12.420	3.71	0.038
Q06787-11	Isoform 11 of Fragile X messenger ribonucleoprotein 1	16.136	12.943	3.19	0.043
A0A499FJF3	Rho-GAP domain-containing protein	12.060	8.908	3.15	0.033
Q8N573	Oxidation resistance protein 1	9.846	6.746	3.10	0.047
Q86YH6	All trans-polyprenyl-diphosphate synthase PDSS2	11.335	8.284	3.05	0.034
Q7Z589	BRCA2-interacting transcriptional repressor EMSY	13.745	10.715	3.03	0.045
P07602	Prosaposin	9.544	6.574	2.97	0.011

**Table 2:** Differentially expressed proteins with Infinity score.

Uniprot Accession	Name	Avg (log_2_)		Log_2_FC	*p* Adjusted
		LRS	DMSO		
Q9Y2M2	Protein SSUH2 homolog	7.46	ND	Infinity	0.019
Q68D51	DENN domain-containing protein 2C	9.85	ND	Infinity	0.023
C9JQV0	Chromosome 7 open reading frame 50	ND	10.93	Infinity	0.008
Q5SRE5	Nucleoporin NUP188	ND	7.69	Infinity	0.010
O95672	Endothelin-converting enzyme-like 1	ND	8.57	Infinity	0.010
P26010	Integrin beta-7	ND	6.26	Infinity	0.010
Q8N1I0	Dedicator of cytokinesis protein 4	ND	8.76	Infinity	0.010
P12757	Ski-like protein	ND	8.36	Infinity	0.021
Q9Y6N6	Laminin subunit gamma-3	ND	8.96	Infinity	0.032
A0A8I5KW95	Heparan sulfate 2-O-sulfotransferase	ND	9.50	Infinity	0.035

ND = Not Detected, indicates that the protein was not present in the sample.

**Table 3:** List of differentially expressed proteins in response to the effect of the LRS combination, tagged with GO terms associated with hallmarks of cancer.

Hallmark of Cancer	Gene of Protein	GO Identifier	GO ID	Description
**SUSTAINING PROLIFERATIVE SIGNALING**	*CUL2*, *EML4*, *INTS13*, *INTS3*, *MAPK7*, *RB1CC1*, *SNX9*, *TRIP13*	9606.ENSP00000362441	GO:0007049	Cell cycle
	*EML4*, *INTS13*, *SNX9*	9606.ENSP00000259008	GO:0051301	Cell division
	*FAP*	9606.ENSP00000369497	GO:0008283	Cell proliferation
	*INTS3*, *TP53BP1*, *TRIP13*	9606.ENSP00000362441	GO:0045786	Negative regulation of cell cycle
	*ANKRD17*, *TCF3*, *WIZ*	9606.ENSP00000216714	GO:0045787	Positive regulation of cell cycle
	*FN1*	9606.ENSP00000481038	GO:0030307	Positive regulation of cell growth
	*FLT4*, *FN1*, *TCF3*, *ZAP70*	9606.ENSP00000432472	GO:0008284	Positive regulation of cell proliferation
**EVADING GROWTH SUPPRESSOR**	*CAPRIN2*, *KIAA0319*	9606.ENSP00000377192	GO:0030308	Negative regulation of cell growth
	*CTBP1*, *DICER1*, *FAP*, *RB1CC1*	9606.ENSP00000381736	GO:0008285	Negative regulation of cell proliferation
	*MAPK7*, *NLRP2*, *PEG10*, *RB1CC1*, *SKIL*, *TBC1D10C*, *WWC3*	9606.ENSP00000265132	GO:0009968	Negative regulation of signal transduction
**RESIST CELL DEATH**	*AARS1*, *FLT4*, *MAPK7*, *OXR1*, *RB1CC1*	9606.ENSP00000261772	GO:0043069	Negative regulation of programmed cell death
	*AARS1*, *CUL2*, *DICER1*, *EPB41L3*, *FAP*, *NLRP1*, *NLRP2*, *OXR1*, *PEG10*, *RB1CC1*, *SKIL*	9606.ENSP00000261772	GO:0012501	Programmed cell death
	*AARS1*, *FAP*, *FLT4*, *MAPK7*, *NLRP1*, *OXR1*, *RB1CC1*, *SKIL*	9606.ENSP00000252699	GO:0043067	Regulation of programmed cell death
**ENABLING REPLICATIVE IMMORTALITY**	*NAT10*	9606.ENSP00000299300	GO:0032204	Regulation of telomere maintenance
	*RPA3*	9606.ENSP00000369497	GO:0000723	Telomere maintenance
	*RPA3*	9606.ENSP00000369497	GO:0032200	Organization of telomeres
**INDUCING ANGIOGENESIS**	*BCAS3*, *CALD1*, *FAP*, *FLT4*, *FN1*	9606.ENSP00000303153	GO:0001525	Angiogenesis
**ACTIVATING INVASION AND METASTASIS**	*BAIAP2L1*, *DST*, *FAP*, *FN1*, *IGSF9B*, *ITGB7*, *ZAP70*	9606.ENSP00000265132	GO:0007155	Cell adhesion
	*DST*, *EPB41L3*, *FN1*	9606.ENSP00000276297	GO:0034330	Cell junction organization
	*DOCK4*, *FAP*, *FN1*, *ITGB7*, *KIAA0319*, *NAV1*, *SYDE2*, *WWC3*, *ZAP70*	9606.ENSP00000346550	GO:0016477	Cell migration
	*CCDC40*, *CFAP43*, *DICER1*, *EPB41L3*	9606.ENSP00000380679	GO:0030030	Cell projection organization
	*BCAS3*, *MAPK7*	9606.ENSP00000252699	GO:0007162	Negative regulation of cell adhesion
	*BCAS3*, *FN1*, *MAPK7*, *ZAP70*	9606.ENSP00000327760	GO:0030155	Regulation of cell adhesion
**GENOME INSTABILITY AND MUTATION**	*TP53BP1*	9606.ENSP00000259008	GO:0031570	DNA integrity Checkpoint
	*EMSY*, *FMR1*, *INTS3*, *MTA1*, *RPA3*, *TP53BP1*, *TRIP13*	9606.ENSP00000216714	GO:0006281	DNA repair
**TUMOR PROMOTING INFLAMMATION**	*MAPK7*, *NLRP1*, *NLRP2*	9606.ENSP00000287647	GO:0050727	Regulation of inflammatory response
**AVOIDING IMMUNE DESTRUCTION**	*ANKRD17*, *CD7*, *H2BC12*, *LYAR*, *NLRP1*, *NLRP2*, *ZAP70*	9606.ENSP00000370745	GO:0006955	Immune response
	*ANKRD17*, *LYAR*, *NLRP1*, *TP53BP1*, *ZAP70*	9606.ENSP00000265132	GO:0050776	Regulation of immune response

**Table 4:** Differentially expressed proteins in response to the treatment of HuH7 cells with the drug combination associated with clinical prognosis in HCC.

Gene	Expression	Prognostic Value
*C7ORF50*, NUP188, ATP6AP2, SNRNP40, SH3GL1, LYAR, HNRNPL, INTS13, TRIP13, RPA3, EIF2A, MAPK7, NAT10, CUL2	Downregulated	Unfavorable
*ITGB7*, FLT4		Favorable
*ECE2*, NCOA6, TPM3, PSAP, TCF3, LSG1, RPS5, VRK2, MRPL37, SF3A3	Up regulated	Unfavorable
*ZAP70*, ANGPTL1, EHHADH		Favorable

**Table 5:** List of the main genes differentially expressed after treatment with the LRS combination.

**Downregulated**					
**Gene**	**Name**	**Avg (log_2_)**		**Log_2_FC**	***p*** **Adjusted**
		**LRS**	**DMSO**		
*HIST1H2BM*	Histone cluster 1, H2bm	5.11	9.7	−4.60	8.72 × 10^−6^
*DKK1*	dickkopf WNT signaling pathway inhibitor 1	3.56	8.09	−4.53	1.10 × 10^−5^
*HIST1H2AB*	Histone cluster 1, H2ab	6.32	9.91	−3.40	9.00 × 10^−6^
*KIF20A*	Kinesin family member 20A	4.44	7.78	−3.33	2.67 × 10^−5^
*CDC25A*	Cell division cycle 25A	2.89	6.17	−3.28	4.00 × 10^−4^
*HIST1H1B*	Histone cluster 1, H1b	7.68	10.9	−3.21	9.00 × 10^−6^
*E2F8*	E2F transcription factor 8	3.14	6.34	−3.20	4.00 × 10^−4^
*PDGFRA*	Platelet-derived growth factor receptor, alpha polypeptide	4.02	7.18	−3.16	2.05 × 10^−5^
*HIST2H4A*	Histone cluster 2, H4a	5.88	9.04	−3.16	2.59 × 10^−5^
*KIAA0101*	Casein kinase 1 gamma 1	3.95	7.1	−3.15	7.00 × 10^−4^
*MKI67*	Marker of proliferation Ki-67	5.49	8.64	−3.15	3.20 × 10^−5^
*NTS*	Neurotensin	2.01	5.16	−3.14	4.00 × 10^−4^
*PLK1*	Polo-like kinase 1	6.92	10.04	−3.12	9.00 × 10^−6^
*HIST1H2BI*	Histone cluster 1, H2bi	3.86	6.98	−3.12	3.66 × 10^−5^
*HIST2H3A*	Histone cluster 2, H3a	5.51	8.61	−3.10	1.91 × 10^−5^
**Upregulated**					
**Gene**	**N** **ame**	**Avg (log_2_)**		**Log_2_FC**	***p*** **Adjusted**
		**LRS**	**DMSO**		
*LAMP3*	Lysosomal-associated membrane protein 3	6.21	3.34	2.86	2.00 × 10^−4^
*ALDH1L2*	Aldehyde dehydrogenase 1 family, member L2	6.2	3.35	2.84	7.00 × 10^−4^
*TCP11L2*	t-complex 11, testis-specific-like 2	6.34	3.5	2.84	1.00 × 10^−4^
*FAM129A*	Family with sequence similarity 129, member A	4.63	1.79	2.83	2.80 × 10^−5^
*PPARGC1A*	Peroxisome proliferator-activated receptor gamma, coactivator 1 alpha	6.15	3.72	2.43	1.00 × 10^−4^
*INHBE*	Inhibin beta E	7.97	5.54	2.42	9.38 × 10^−5^
*ASS1*	Argininosuccinate synthase 1	7.26	4.89	2.37	6.76 × 10^−5^
*MT1F*	Metallothionein 1F	8.35	6.02	2.33	3.00 × 10^−4^
*FUCA1*	Fucosidase, alpha-L-1, tissue	6.51	4.21	2.30	6.00 × 10^−4^
*ACSS2*	Acyl-CoA synthetase short-chain family member 2	6.22	3.95	2.27	6.00 × 10^−4^
*SULT1C2*	Sulfotransferase family 1C member 2	5.78	3.52	2.26	4.00 × 10^−4^
*SLC25A36*	Solute carrier family 25 (pyrimidine nucleotide carrier), member 36	7.94	5.71	2.26	4.00 × 10^−4^
*SLC46A3*	Solute carrier family 46, member 3	4.23	2.04	2.22	5.30 × 10^−3^
*HERPUD1*	Homocysteine-inducible, endoplasmic reticulum stress-inducible, ubiquitin-like domain member 1	8.37	6.19	2.18	4.16 × 10^−5^
*INSIG1*	insulin induced gene 1	7.04	4.93	2.10	2.00 × 10^−4^

**Table 6:** Top 15 main signaling pathways altered in response to the LRS combination in HuH7 cells.

**Downregulated Signaling Pathways**		
Signaling Pathway	***p*** **-Adjusted**	**z-Score**
Cell Cycle Checkpoints	9.00 × 10^−80^	−11.49
Mitotic Metaphase and Anaphase	1.50 × 10^−41^	−9.22
DNA Replication Pre-Initiation	7.00 × 10^−60^	−9.05
TCF dependent signaling in response to WNT	1.51 × 10^−35^	−8.61
Deubiquitination	1.21 × 10^−30^	−8.5
Telomere Maintenance	2.07 × 10^−51^	−8.43
Transcriptional regulation by RUNX1	2.68 × 10^−34^	−8.31
Mitotic Prophase	5.51 × 10^−44^	−8.07
HDR through Homologous Recombination (HRR) or Single Strand Annealing (SSA)	9.98 × 10^−41^	−8
Chromatin modifying enzymes	1.63 × 10^−20^	−8
Synthesis of DNA	7.01 × 10^−42^	−7.94
ESR-mediated signaling	1.19 × 10^−32^	−7.81
Meiotic recombination	6.63 × 10^−46^	−7.55
Gene Silencing by RNA	8.42 × 10^−36^	−7.55
Oxidative Stress Induced Senescence	1.15 × 10^−32^	−7.42
**Upregulated signaling pathway**		
Granzyme A Signaling	4.38 × 10^−8^	3.64
GAIT Translation Signaling Pathway	0.0205	3.15
Metallothioneins bind metals	6.48 × 10^−9^	3
Cholesterol biosynthesis	0.00013	3
Superpathway of Cholesterol Biosynthesis	0.00018	3
ATF4 activates genes in response to endoplasmic reticulum stress	0.00085	2.83
Coronavirus Pathogenesis Pathway	0.00037	2.56
Mitochondrial Dysfunction	0.00024	2.48
Cholesterol Biosynthesis I	0.00039	2.45
Cholesterol Biosynthesis II (via 24,25-dihydrolanosterol)	0.00039	2.45
Cholesterol Biosynthesis III (via Desmosterol)	0.00039	2.45
Parkinson’s Signaling Pathway	0.00145	1.89
Activation of gene expression by SREBF (SREBP)	3.66 × 10^−5^	1.73
Cell Cycle: G2/M DNA Damage Checkpoint Regulation	3.84 × 10^−8^	1.6
Sumoylation Pathway	0.01497	1.51

**Table 7:** List of differentially expressed genes in response to the effect of the LRS combination, tagged with GO terms associated with hallmarks of cancer.

Hallmark of Cancer	DEGs	GO Identifier	GO ID	Description
*Sustaining proliferative signaling*	DDIT3, FEN1, H2AFX, KIF15, RCC2	9606.ENSP00000448665	GO:0007049	Cell cycle
	RCC2	9606.ENSP00000259008	GO:0051301	Cell division
	DDIT3	9606.ENSP00000369497	GO:0008283	Cell proliferation
	H2AFX	9606.ENSP00000362441	GO:0045786	Negative regulation of cell cycle
	FEN1, RCC2	9606.ENSP00000216714	GO:0045787	Positive regulation of cell cycle
	NMB	9606.ENSP00000432472	GO:0008284	Positive regulation of cell proliferation
	CPB2	9606.ENSP00000381736	GO:0008285	Negative regulation of cell proliferation
*Evading growth suppressor*	DDIT3	9606.ENSP00000265132	GO:0009968	Negative regulation of signal transduction
*Resist cell death*	DDIT3, ZDHHC3	9606.ENSP00000261772	GO:0012501	Programmed cell death
	DDIT3, KNG1	9606.ENSP00000252699	GO:0043067	Regulation of programmed cell death
	H2AFX	9606.ENSP00000369497	GO:0090398	Cellular senescence
*Enabling replicative immortality*	FEN1	9606.ENSP00000369497	GO:0000723	Telomere maintenance
	FEN1	9606.ENSP00000369497	GO:0032200	Telomere organization
*Inducing angiogenesis*	RHOB	9606.ENSP00000248553	GO:0045766	Positive regulation of angiogenesis
	CLIC1, RCC2	9606.ENSP00000265132	GO:0007155	Cell adhesion
*Activating invasion and metastasis*	RCC2, WASF1	9606.ENSP00000276297	GO:0034330	Cell junction Organization
	DDIT3	9606.ENSP00000346550	GO:0016477	Cell migration
	WASF1	9606.ENSP00000380679	GO:0030030	Cell projection Organization
	KNG1, RCC2	9606.ENSP00000252699	GO:0007162	Negative regulation of cell adhesion
	KNG1, RCC2	9606.ENSP00000327760	GO:0030155	Regulation of Cell Adhesion
	H2AFX	9606.ENSP00000259008	GO:0031570	DNA integrity Checkpoint
*Genome instability and mutation*	FEN1, H2AFX	9606.ENSP00000216714	GO:0006281	DNA repair
	KNG1, NMB	9606.ENSP00000370745	GO:0006955	Immune Response

**Table 8:** Differentially expressed genes in response to treatment with the LRS combination are involved in signaling pathways relevant to cancer and associated with poor clinical prognosis in HCC.

Biological Process	DEGs	Clinical Value
**Cell Cycle**	*PLK1*, *AURKB*, *BUB1*, *CDCA8*, *CCNA2*, *MCM7*, *CCNB2*, *CDC20*, *CCNB1*, *GTSE1*, *KIF2C*, *CDC45*, *INCENP*, *NDC80*, *ORC1*, *CDK2*, *MCM10*, *CDC25A*, *CDC25C*, *MCM8*, *CDC6*, *MCM3*, *ZWINT*, *DYNLL1*, *MCM4*, *RPA1*, *MCM5*, *CDK1*, *SKA2*, *CENPO*, *UBE2C*, *ADRM1*, *CHEK1*, *MAD2L1*, *PSMB3*, *PSME3*, *BUB1B*, *PSMD11*, *UBE2E1*, *PSMD1*, *DNA2*, *PSMD2*, *NUDC*, *RFC3*, *PSMB4*, *SKA1*, *SPC25*, *CENPI*, *PSMA3*, *PSMA7*, *BLM*, *CENPM*, *RHNO1*, *PSMD3*, *RANGAP1*, *PSMA2*, *MCM6*, *EXO1*, *PSMD14*, *NUF2*, *PSMB1*, *YWHAQ*, *PSMD13*, *PKMYT1*, *SPDL1*, *ZWILCH*, *PSMB5*, *PSMA5*, *BIRC5*, *CKAP5*, *UBE2S*, *MCM2*, *CENPN*, *PSMC1*, *CENPF*, *RPA3*, *CDKN2A*, *KIF18A*, *DBF4*, *BRCA1*, *ORC6*, *NUP85*, *ATRIP*, *CENPA*, *PSMC5*, *CENPH*, *RFC2*	Unfavorable
**DNA Replication**	*MCM7*, *CDT1*, *ORC1*, *MCM8*, *CDC6*, *MCM3*, *MCM4*, *MCM5*, *UBE2C*, *ADRM1*, *PSMB3*, *PSME3*, *PSMD11*, *UBE2E1*, *PSMD1*, *PSMD2*, *PSMB4*, *PSMA3*, *PSMA7*, *PSMD3*, *PSMA2*, *MCM6*, *PSMD14*, *PSMB1*, *PSMD13*, *PSMB5*, *PSMA5*, *UBE2S*, *MCM2*, *PSMC1*, *ORC6*, *PSMC5*	Unfavorable
**DNA Synthesis**	*GINS1*, *CCNA2*, *MCM7*, *CDC45*, *CDT1*, *ORC1*, *PCNA*, *PRIM1*, *CDK2*, *MCM8*, *CDC6*, *MCM3*, *GINS2*, *MCM4*, *RPA1*, *POLA1*, *MCM5*, *FEN1*, *GINS4*, *UBE2C*, *ADRM1*, *PSMB3*, *PSME3*, *PSMD11*, *UBE2E1*, *PSMD1*, *DNA2*, *PSMD2*, *RFC3*, *POLE2*, *PSMB4*, *PRIM2*, *PSMA3*, *PSMA7*, *SKP2*, *PSMD3*, *PSMA2*, *MCM6*, *PSMD14*, *PSMB1*, *PSMD13*, *POLD2*, *POLA2*, *PSMB5*, *PSMA5*, *UBE2S*, *MCM2*, *PSMC1*, *RPA3*, *ORC6*, *POLE4*, *PSMC5*, *RFC2*	Unfavorable

**Table 9:** Metabolites identified as significantly differentially abundant in HuH7 cells after treatment with LRS combination.

**Less Abundant Metabolites**				
**Metabolite**	**Avg (log** **_2_** **)**		**Log** **_2_** **FC**	***p*** **Adjusted**
	**LRS**	**DMSO**		
**Creatine**	18.36	21.26	−2.9074	2.84 × 10^−4^
**Phosphocholine**	22.62	24.03	−1.4111	8.46 × 10^−4^
**Lactate**	23.58	25.96	−2.3846	2.70 × 10^−5^
**Alanine**	21.28	23.54	−2.2608	8.98 × 10^−6^
**Acetate**	21.72	23.13	−1.4107	0.003029
**Glutamate**	22.17	24.00	−1.8373	8.98 × 10^−6^
**Most Abundant Metabolites**				
**Metabolite**	**Avg (log** **_2_** **)**		**Log** **_2_** **FC**	***p*** **Adjusted**
	**LRS**	**DMSO**		
**Glucose**	22.56	21.17	1.4069	0.001767
**Fructose**	22.22	20.43	1.7903	0.010317
**Aspartate**	20.35	17.83	2.4475	0.031021

**Table 10:** Representative PPI enrichment pathways by clusters.

Cluster	Description	*p*-Adjusted Value	Genes
1	Mixed, incl. Amplification of signal from the kinetochores, and Condensin complex	0.00051	*TRIP13*, *NDC80*, *KIF2C*
	Regulation of chromosome segregation	0.0045	*TRIP13*, *NDC80*, *KIF2C*
	Nuclear division	0.0126	*TRIP13*, *NDC80*, *KIF2C*
	Female meiosis I	0.0126	*TRIP13*, *NDC80*
	Attachment of mitotic spindle microtubules to kinetochore	0.0126	*NDC80*, *KIF2C*
	Nuclear chromosome segregation	0.0126	*TRIP13*, *NDC80*, *KIF2C*
	Mitotic spindle assembly checkpoint signaling	0.0142	*TRIP13*, *NDC80*
	Mitotic cell cycle process	0.0142	*TRIP13*, *NDC80*, *KIF2C*
	Mixed, incl. Regulation of mitotic sister chromatid segregation, and Kinesin motor domain, conserved site	0.0162	*TRIP13*, *KIF2C*
	Mixed, incl. Amplification of signal from the kinetochores, and Condensin complex	0.00051	*TRIP13*, *NDC80*, *KIF2C*
2	Termination of translesion DNA synthesis	0.00000002	*RPA3*, *PCNA*, *RFC5*, *USP10*
	Translesion synthesis by REV1	0.000002	*RPA3*, *PCNA*, *RFC5*
	Translesion Synthesis by POLH	0.000002	*RPA3*, *PCNA*, *RFC5*
	PCNA-Dependent Long Patch Base Excision Repair	0.000002	*RPA3*, *PCNA*, *RFC5*
	Translesion synthesis by POLK	0.000002	*RPA3*, *PCNA*, *RFC5*
	Translesion synthesis by POLI	0.000002	*RPA3*, *PCNA*, *RFC5*
	Lagging Strand Synthesis	0.000002	*RPA3*, *PCNA*, *RFC5*
	Gap-filling DNA repair synthesis and ligation in GG-NER	0.000002	*RPA3*, *PCNA*, *RFC5*
	Mismatch repair	0.000003	*RPA3*, *PCNA*, *RFC5*
	Recognition of DNA damage by PCNA-containing replication complex	0.000003	*RPA3*, *PCNA*, *RFC5*
3	Cholesterol biosynthesis pathway	0.0000005	*SQLE*, *HMGCS1*, *DHCR7*
	Cholesterol synthesis disorders	0.0000005	*SQLE*, *HMGCS1*, *DHCR7*
	Cholesterol metabolism	0.00000187	*SQLE*, *HMGCS1*, *DHCR7*
	Cholesterol metabolism with Bloch and Kandutsch-Russell pathways	0.000003	*SQLE*, *HMGCS1*, *DHCR7*
	Cholesterol biosynthesis	0.00000727	*SQLE*, *HMGCS1*, *DHCR7*
	Cholesterol metabolism	0.00000828	*SQLE*, *HMGCS1*, *DHCR7*
	Activation of gene expression by SREBF (SREBP)	0.0000127	*SQLE*, *HMGCS1*, *DHCR7*
	Sterol biosynthetic process	0.00021	*SQLE*, *HMGCS1*, *DHCR7*
	Steroid biosynthesis	0.0012	*SQLE*, *DHCR7*
	Cholesterol metabolic process	0.0017	*SQLE*, *HMGCS1*, *DHCR7*

**Table 11:** Survival analysis and prognostic impact of gene clusters.

Cluster	*p*-Value	Hazard Ratio (±95% CI)	High Median Survival	Low Median Survival	Effect	Interpretation
Cluster 1: *NDC80*, *KIF2C*, *TRIP13*	0.00016	2.23 (1.26–3.95)	15.42	58.88	High worse	Risk
Cluster 2: *RPA3*, *RFC5*, *USP10*, *PCNA*	0.033	1.56 (0.97–2.5)	25.25	58.88	High worse	Risk
Cluster 3: *SQLE*, *DHCR7*, *HMGCS1*	0.753	1.14 (0.72–1.79)	55.69	55.40	NS	NS

CI: Confidence Interval; NS Not significant. Median survival is expressed in months; statistical significance was evaluated using log-rank test.

## Data Availability

The analyzed and raw data from the current study are available from the corresponding author upon request.

## Supplementary Materials

The following supporting information can be downloaded at: https://www.mdpi.com/article/10.3390/biomedicines14091898/s1. File S1. Total number of proteins differentially expressed in response to treatment with the LRS combination in HuH7 cells; File S2. Total number of transcripts differentially expressed in response to treatment with the LRS combination in HuH7 cells; File S3. Functional enrichment analysis of differentially expressed genes in HuH7 cells treated with LRS combination; File S4. Cross-omics correlation analysis between mRNA and protein expression changes; File S5. Multivariate Cox proportional hazards analysis in TCGA cohort; File S6. Basal transcript expression profiles of the target gene cluster across hepatocellular carcinoma cell lines; File S7. Basal relative protein expression profiles of the target cluster components across hepatocellular carcinoma cell lines; File S8. Essentiality profiles and CRISPR-Cas9 dependency scores of target genes across hepatocellular carcinoma cell lines; File S9. Genomic and transcriptomic alteration landscape of the 5-gene cluster in primary hepatocellular carcinoma patients; File S10. Cross-omics validation and concordance analysis between proteomic profiles and transcriptomic expression levels.

## Abbreviations

The following abbreviations are used in this manuscript:

*C7orf50*	Chromosome 7 open reading frame 50
DEGs	Differentially expressed genes
GO	Gene Ontology
HCC	Hepatocellular carcinoma
HPA	Human protein atlas
*HS2ST1*	Heparan sulfate 2-O-sulfotransferase 1
IPA	Ingenuity Pathway Analysis
KEGG	Kyoto Encyclopedia of Genes and Genomes
*KIF2C*	Kinesin family member 2C
LIHC	Liver Hepatocellular Carcinoma
LRS	Loratadine, raloxifene, sorafenib
*NDC80*	Kinetochore complex component
NMR	Nuclear Magnetic Resonance
*NUP188*	Nucleoporin 188
ORA	Over-representation analysis
PCA	Principal component analysis
PCNA	proliferating cell nuclear antigen
PPI	Protein–protein interaction
*RPA3*	Replication protein A3
TAC	Transcriptome Analysis Console
TCGA	The Cancer Genome Atlas
*TRIP13*	Thyroid hormone receptor interactor 13
